# Spatial multiplex analysis of lung cancer reveals that regulatory T cells attenuate KRAS-G12C inhibitor–induced immune responses

**DOI:** 10.1126/sciadv.adl6464

**Published:** 2024-11-01

**Authors:** Megan Cole, Panayiotis Anastasiou, Claudia Lee, Xiaofei Yu, Andrea de Castro, Jannes Roelink, Chris Moore, Edurne Mugarza, Martin Jones, Karishma Valand, Sareena Rana, Emma Colliver, Mihaela Angelova, Katey S. S. Enfield, Alastair Magness, Asher Mullokandov, Gavin Kelly, Tanja D. de Gruijl, Miriam Molina-Arcas, Charles Swanton, Julian Downward, Febe van Maldegem

**Affiliations:** ^1^ Oncogene Biology Laboratory, Francis Crick Institute, London, UK.; ^2^ Cancer Evolution and Genome Instability Laboratory, Francis Crick Institute, London, UK.; ^3^ Department of Molecular Cell Biology and Immunology, Amsterdam UMC, Vrije Universiteit Amsterdam, Amsterdam, Netherlands.; ^4^ Cancer Center Amsterdam, Cancer Biology and Immunology, Amsterdam, Netherlands.; ^5^ Amsterdam Institute for Immunology and Infectious Diseases, Amsterdam, Netherlands.; ^6^ Electron Microscopy, Francis Crick Institute, London, UK.; ^7^ Bioinformatics and Biostatistics, Francis Crick Institute, London, UK.; ^8^ Department of Medical Oncology, Amsterdam UMC, Vrije Universiteit Amsterdam, Amsterdam, Netherlands.; ^9^ Cancer Research UK Lung Cancer Centre of Excellence, UCL Cancer Institute, London, UK.

## Abstract

Kirsten rat sarcoma virus (KRAS)–G12C inhibition causes remodeling of the lung tumor immune microenvironment and synergistic responses to anti–PD-1 treatment, but only in T cell infiltrated tumors. To investigate mechanisms that restrain combination immunotherapy sensitivity in immune-excluded tumors, we used imaging mass cytometry to explore cellular distribution in an immune-evasive KRAS mutant lung cancer model. Cellular spatial pattern characterization revealed a community where CD4^+^ and CD8^+^ T cells and dendritic cells were gathered, suggesting localized T cell activation. KRAS-G12C inhibition led to increased PD-1 expression, proliferation, and cytotoxicity of CD8^+^ T cells, and CXCL9 expression by dendritic cells, indicating an effector response. However, suppressive regulatory T cells (T_regs_) were also found in frequent contact with effector T cells within this community. Lung adenocarcinoma clinical samples showed similar communities. Depleting T_regs_ led to enhanced tumor control in combination with anti–PD-1 and KRAS-G12C inhibitor. Combining T_reg_ depletion with KRAS inhibition shows therapeutic potential for increasing antitumoral immune responses.

## Teaser

Spatial analysis identified T_regs_ as a source of local T cell repression, mediating resistance to KRAS-G12C-i and anti–PD-1.

## INTRODUCTION

Recent years have seen a transformation in the treatment of non–small cell lung cancer (NSCLC), with the introduction of immune checkpoint blockade, which has increased survival rates of patients with a previously poor prognosis. Despite this, only a subset of patients responds, and many responders acquire resistance over time (*1*, *2*). In 2021, a further breakthrough occurred when the Kirsten rat sarcoma virus (KRAS) inhibitor sotorasib was approved for the treatment of locally advanced or metastatic KRAS-G12C mutant NSCLC. This followed successful clinical trials where 80% of patients achieved temporary disease control following sotorasib treatment. However, despite a modest improvement in progression-free survival, sotorasib gave no improvement in overall survival compared to docetaxel (*3*), demonstrating its limitations for use as a monotherapy. Therefore, strategies for combination with other therapies are being urgently sought (*4*, *5*).

The importance of the immune system in the response to KRAS-G12C inhibition was revealed when Canon *et al.* (*6*) showed that T cell presence was essential for durable responses in subcutaneous tumors of the colon cancer model CT26 treated with sotorasib. In addition, Briere *et al.* (*7*) using the same model demonstrated a switch in the tumor microenvironment (TME) from immunosuppressive in the vehicle setting, with high presence of M2 macrophages and myeloid-derived suppressor cells, to favoring antitumoral immune response following KRAS-G12C inhibition with adagrasib, another clinically approved KRAS-targeted drug. CT26 tumors show an immune hot TME (i.e., high T cell infiltration rates) and are responsive to single-agent immune checkpoint inhibition (ICI). Similar to the proinflammatory responses observed in this colon cancer model (*6*, *7*), our previous work also revealed remodeling of the TME following KRAS-G12C inhibition in multiple lung tumor models, including the immune cold orthotopic Lewis lung murine NSCLC tumor model that was genetically engineered to disrupt the NRAS gene, avoiding redundancy in RAS signaling (3LL ΔNRAS, here referred to as 3LL in short) (*8*, *9*). We found that oncogenic KRAS suppresses interferon signaling within the tumor cells via MYC, leading to a proinflammatory cascade upon KRAS inhibition (*9*). KRAS-G12C inhibitors were also shown to synergize well with ICI in lung cancer models, but only in immune hot TME settings (*8*, *10*). These findings highlight that while KRAS-G12C inhibitors specifically target tumor cells, this results in profound secondary effects on the TME and T cells are crucial for durable responses.

Our previous analysis established that following seven consecutive days of treatment with the KRAS-G12C inhibitor MRTX1257, although 3LL tumor growth was inhibited, tumors did not regress, indicating that KRAS-G12C inhibitors alone are not sufficient to cause tumor regression in this model (*9*). Combination of this KRAS-G12C inhibitor with anti–programmed cell death protein 1 (PD-1) therapy did not increase responses in this model (*8*). This is reflective of the failure to see a beneficial effect on response of combined KRAS inhibition and PD-1 blockade in the clinical setting, either due to combination toxicities or lack of efficacy (*11*). The unmet need for new combinatorial treatment options that improve T cell–mediated antitumoral immune responses in immune cold tumor models has hence become increasingly clear. We therefore used the 3LL immune-evasive orthotopic lung tumor model, in which effector immune cells are excluded from the tumor, to seek more effective therapeutic combinations with KRAS-G12C inhibition.

We used imaging mass cytometry (IMC) to analyze the makeup of these tumors in situ. This method is particularly useful for study of the TME due to its ability to capture up to 40 markers simultaneously. Spatial information remains intact, meaning that cell phenotypes can be analyzed in the context of their spatial neighbors (*12*). Obtaining spatial information is essential when studying the TME, as it provides insight into the cellular interactions dictating local immune activation or suppression, as mechanisms of immune response or resistance to treatment, and has been shown to improve prediction of clinical outcomes compared to cell frequency alone in patients with NSCLC (*13*).

Here, we present data on the identification of neighborhood communities through single-cell spatial analysis to investigate which cellular interaction patterns may restrain antitumoral immune responses in the 3LL tumor model. A community resembling a T cell activation hub was identified, where regulatory T cell (T_reg_) interactions may play a key role in dampening antitumoral immune responses following KRAS-G12C inhibition. In parallel, cellular community analysis of treatment-naïve human lung adenocarcinoma (LUAD) patient samples from the TRAcking Cancer Evolution through therapy (Rx) (TRACERx) IMC cohort (*14*) suggested that similar local T_reg_ control may be restraining immune responses in a subset of patients.

Together, this led us to explore the effect of combining KRAS-G12C inhibitor with a T_reg_ depleting anti–cytotoxic T lymphocyte–associated protein 4 (CTLA-4) antibody in the in vivo orthotopic setting, where markedly improved responses were noted. This opens up the perspective of combining KRAS-G12C inhibitors with T_reg_ targeting to improve durable response rates.

## RESULTS

### Introduction to spatial communities and validation

Our previous IMC analyses on 3LL lung tumors have shown that there are clear patterns in the arrangements of cells in the lung cancer tissues and changes to those patterns occur in response to KRAS-G12C inhibition (*9*). For example, we saw one subset of macrophages lining the tumor-normal interface, while another type of macrophages was intermixed with the tumor cells. Effector cells such as T cells and B cells were excluded from the tumor domain, while treatment with KRAS-G12C inhibitor MRTX1257 induced movement of T cells and antigen-presenting cells into the tumor domain. We also noted that the phenotypes of cells differed depending on their location in the tissue and hypothesized that the local neighborhood is likely to strongly influence the activation or inhibition of immune cells. Therefore, we adopted a cellular community analysis to cluster cells based on the composition of their local neighborhood [adapted with modifications from (*15*)]. Analysis focused on two previously published datasets (*8*, *9*), both generated from an in vivo experiment in which the Lewis lung (3LL) carcinoma model was treated with the KRAS-G12C inhibitor MRTX1257 or vehicle for 7 days before harvesting of the lungs. The tumors were stained with two partially overlapping antibody panels to give two datasets, with the dataset 2 panel being more T cell oriented (fig. S1, A and B). The cell typing was derived from our previous analyses and based on lineage markers only but independent of maturation or activation markers. Notably, the subsequent cellular communities were therefore blind to cell phenotypes.

**Fig. 1.**
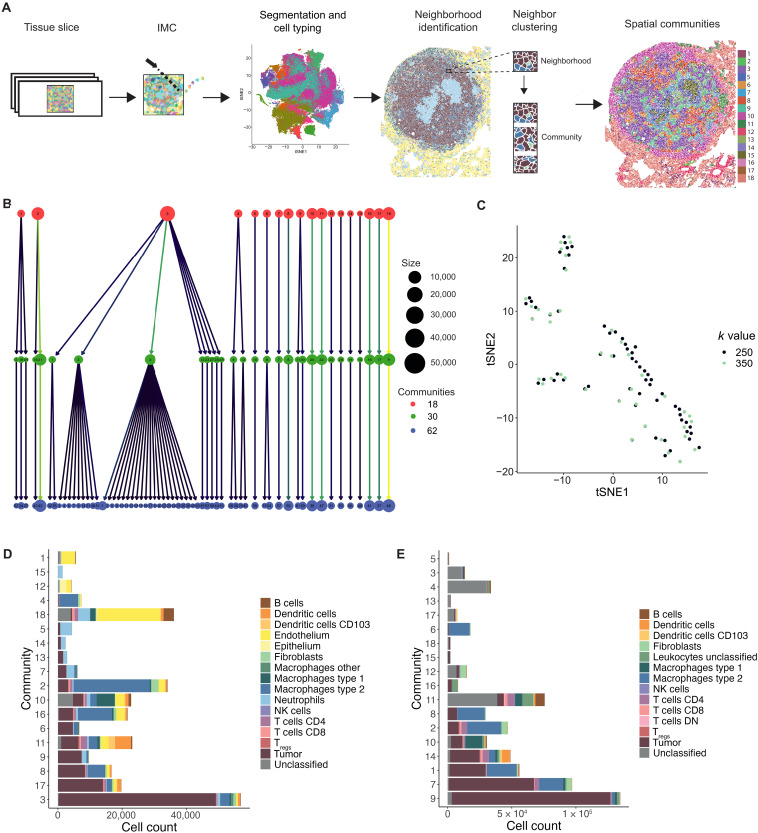
Clustering of cells based on their neighbors yield spatial communities. (**A**) Workflow of generating spatial communities using the data generated from lung tissue slices. (**B**) Cluster tree of 62 communities generated using Rphenograph with a *k*-input value of 250, agglomerated to 30 communities and subsequently agglomerated again to 18 communities, where each circle represents a community and lines indicate communities that were merged during agglomeration. (**C**) tSNE plot of 62 communities generated with a *k*-input value of 250 and 47 communities generated with a *k*-input value of 350 into Rphenograph using dataset 1, where tSNE analysis was run on the basis of the proportion of each cell type contributing to each community. (**D** and **E**) Eighteen spatial communities generated from clustering based on neighbor proportions of each cell type for (D) dataset 1 and (E) dataset 2, with the size of each bar representing cell count of that community and colors indicating the contribution of each cell type. Bars are ordered by decreasing tumor cell count. NK, natural killer; DN, double negative.

We identified neighbors by a 15-pixel expansion of the cell boundary through segmentation in CellProfiler. A 15-pixel radius was chosen as it depicts the average size of a cell, and, therefore, cells identified as neighbors would be those “up to one cell away.” As a result, neighborhoods were not equal in cell number but, instead, reflected the local density surrounding each cell. Louvain clustering using Rphenograph was then run on the neighbor proportion information per cell to identify recurring spatial patterns in the tissue, labeled “spatial communities” or just “communities” in short (Fig. 1A). Graph building with a *k*-nearest neighbor input value of 250 yielded 62 communities for dataset 1; these were agglomerated to 30 and subsequently 18 communities. Agglomeration merged the communities with the lowest cell diversity, representing mainly small variations in the tumor cell neighbors (e.g., agglomerated community 3), while the highly diverse communities, such as those with a high proportion of immune cells, remained stable (Fig. 1B and fig. S1C). We were most interested in the immune-dominated communities for this analysis and therefore decided that 18 communities were optimal to carry forward for our investigation into antitumoral immune response.

To determine whether our method to identify spatial communities was robust to altered clustering input conditions, we also ran Rphenograph clustering on the neighborhood information for all cells in dataset 1 with a *k*-input value of 350. Dimensionality reduction using *t*-distributed stochastic neighbor embedding (tSNE) of the 62 communities identified from *k*-input value of 250 and the 47 communities identified from a *k*-input value of 350 revealed very similar patterns of community phenotypes (Fig. 1C). In addition, there were multiple overlaps between communities identified using the different *k* values for clustering, suggesting matching phenotypes.

The 254 communities identified from neighbor clustering of dataset 2 using a *k*-input of 250 were also agglomerated to 18 communities to enable parallel analysis with the communities identified in dataset 1. The communities in both datasets varied largely in size, with some containing fewer than 2000 cells and others comprising over 50,000 cells (Fig. 1, D and E). There was also differing heterogeneity of these spatial groups, with some being dominated by a single cell type, such as tumor cells in community 3 from dataset 1 and community 9 from dataset 2, while others comprised a mixture of various cell types in more balanced proportions (fig. S1, D and E).

Datasets 1 and 2 were based on different antibody panels and therefore not identifying all the same cell types. For example, lack of markers epithelial cellular adhesion molecule and platelet endothelial cell adhesion molecule-1 for dataset 2 meant that endothelial and epithelial cells could not be identified and, thus, a large number of cells were labeled as “unclassified.” Nevertheless, a separate community clustering analysis on both datasets based on shared cell types only demonstrated that the method to generate communities was stable to altered input data (fig. S1F).

### Tissue architecture reflected in spatial communities

**Fig. 2.**
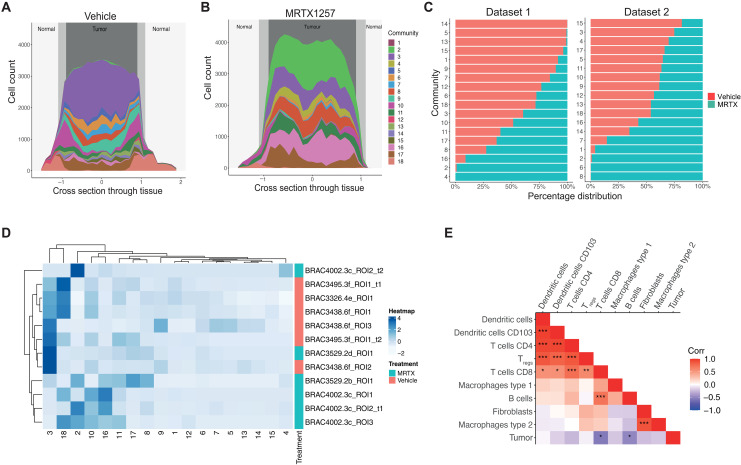
Spatial communities reflect refined tissue architecture and have functional relevance. (**A** and **B**) Cell count per community relative to cross section through the tissue, where 0 represents the center point of the tumor in (A) vehicle and (B) MRTX1257 treatment settings. (**C**) Percentage distribution of each community across vehicle (left) and MRTX1257 (right) treatment groups. Bars are ordered by increasing percentage distribution in vehicle setting. (**D**) Hierarchical clustering of community proportion per ROI for dataset 1, with the use of dendrograms to show relationships between similar ROIs, similar communities, and community distribution across the treatment groups. (**E**) Pearson correlation calculation on the proportion of each cell type pair within each community. *P < 0.05, **P < 0.01, and ***P < 0.001. Cell types were clustered on the basis of correlation value. MRTX, MRTX1257.

As the spatial communities are based on local neighborhoods but these local neighborhoods are likely to be different within different regions of the tissue, we further explored the link with tissue architecture. For dataset 1, we also had information about the three tissue domains that each cell had been assigned to during image segmentation, i.e., tumor, normal, and interface (*9*). As the communities had a nonrandom distribution across the domains, we visualized the distribution of each community relative to the cross section through the tissue to further expand on this spatial organization of the communities (Fig. 2A). For example, in the vehicle setting, community 18, with high endothelium and B cell portion, was restricted to the normal nontumor region as its cell count diminished going into the tumor bulk (fig. S2A). In addition, community 10, with high type 1 macrophage contribution, peaked in cell count at the tumor boundary, demonstrating a clear interface region between normal tissue and the tumor bulk (fig. S2B). There were also further communities, numbers 2 and 3, that were found only in the tumor region, albeit at very different frequencies between vehicle and MRTX1257 conditions (fig. S2, C and D).

We previously observed that following treatment with MRTX1257, the immune-excluded phenotype of this tumor model was remodeled into a more inflammatory immune–infiltrated TME (*9*). Here, we could see that this conversion was also reflected in spatial distribution of the communities, suggesting that tissue domain definition was lost following KRAS-G12C inhibition. Not only did the concentration of communities such as 10 and 18 to the interface and normal regions become less pronounced, but also spatial patterns within the tumor bulk changed following KRAS-G12C inhibition, as the frequency of many communities, such as 2 (type 2 macrophage dominant), 3 (tumor dominant), and 16 (mixed phenotype with high type 2 macrophage portion) was altered, suggesting a transition to a new organization of the TME (Fig. 2B and fig. S2E).

Comparing the relative contribution of each community between the vehicle and MRTX1257 treatment groups revealed that prevalence of many spatial patterns was altered following KRAS-G12C inhibition. Some of these shifts in communities captured changes that we previously described at the single-cell level (*9*). For example, communities 5, 13, 14, and 15 from dataset 1 were found solely in tumors treated with vehicle. These communities represent abundant interactions between tumor cells and neutrophils, which were lost or dispersed following treatment with MRTX1257 (Fig. 2C and fig. S1D). This is in line with a previous observation that the number of neutrophils within the tumor domain was substantially reduced following treatment (*9*). Alternatively, communities 2, 4, and 16 from dataset 1 and communities 2, 6, and 8 from dataset 2 were almost exclusively found in tumors following treatment with MRTX1257. A shared feature for these communities was a neighborhood involving high numbers of type 2 macrophages (F4/80^+^ CD206^+^), which we previously showed to become greatly increased in number upon KRAS-G12C inhibition (*9*). This agreement with previous observations suggests that the communities are reflecting relevant biological processes. Further supporting this notion was the ability to largely separate tissues into the two treatment groups when clustering the frequency of each spatial community per region of interest (ROI) (Fig. 2D and fig. S2F).

One way to infer potential cellular relationships is by correlating cell frequencies, measuring co-occurrences. Therefore, we calculated the correlation of each cell type pair within communities. Strong positive correlations were seen between certain cell types, such as T cells among themselves, T cells with dendritic cells (DCs), type 2 macrophages with fibroblasts, and CD8^+^ T cells with B cells when calculated per community (Fig. 2E and fig. S2G). By contrast, a number of these, such as T_regs_ with CD4^+^and CD8^+^ T cells, and most T cell–DC relationships showed lack of significance when quantified in the ROIs (fig. S2, H and I). This demonstrates the benefit of studying cellular relationships through the identification of localized spatial patterns, as they provide increased statistical power about the interactions occurring in the TME in comparison to measuring interaction of cells across a whole tissue.

### Spatial communities that are abundant in CD8^+^ T cells

Because the abundance of CD8^+^ T cells is associated with positive outcomes in relation to antitumoral immune response, we decided to investigate the T cell–rich communities from this tumor model. Previous analysis revealed increased numbers of CD8^+^ T cells inside the tumor domain following KRAS-G12C inhibition in this model (*9*).

**Fig. 3.**
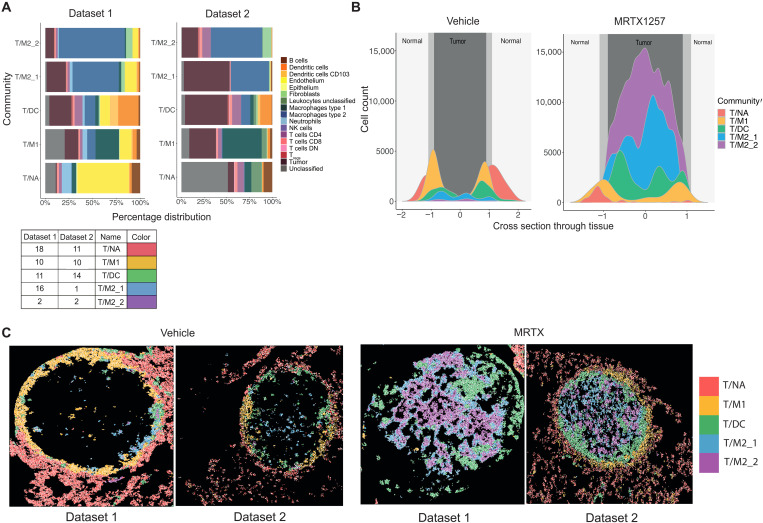
Spatial communities that are abundant in CD8^+^ T cells are diverse in composition and spatial distribution. (**A**) Percentage distribution of cell types contributing to the top five communities with the highest CD8^+^ T cell count from dataset 1 (left) and dataset 2 (right), ordered in pairs based on proportions of shared cell types and labeled with the names T/NA, T/M1, T/DC, T/M2_1, and T/M2_2. (**B**) Cell count of each of the top five communities relative to the cross section through the tissue, where 0 represents the center point of the tumor. (**C**) Visualization of cell outlines from cells assigned to T/NA, T/M1, T/DC, T/M2_1, and T/M2_2 communities, with each outline filled with a color, associating it to one of the five communities, in vehicle- and MRTX1257-treated tumors for datasets 1 and 2.

The top five communities with the highest CD8^+^ T cell count were identified from dataset 1 and dataset 2 (fig. S3, A and B). These five CD8^+^ T cell–rich communities from both datasets paired up phenotypically, particularly when only shared cell types between datasets were considered (fig. S3C). Therefore, unique colors and the names T cell/normal adjacent community (T/NA), T cell/type 1 macrophage community (T/M1), T cell/DC community (T/DC), T cell/type 2 macrophage community 1 (T/M2_1), and T cell/type 2 macrophage community 2 (T/M2_2) were assigned to each pair for parallel analysis going forward (Fig. 3A). These five communities contained over 75% of the total CD8^+^ T cell population from both datasets, suggesting a representative population of the overall cohort (fig. S3D). These communities had widely different compositions, placing the T cells in very diverging spatial contexts (Fig. 3A). The T/NA community was characterized by high endothelial cell content; in the T/M1 community, the type 1 macrophages (CD11c^+^ CD68^+^) (*9*) were the most abundant cell type; the T/DC community was strongly enriched in various T cell subsets and DCs; and T/M2_1 and T/M2_2 communities were dominated by tumor cells and type 2 macrophages (F4/80^+^ CD206^+^) (*9*) in different ratios.

These CD8^+^ T cell–rich communities also differed in presence between treatment groups. The T/NA community was more frequently found following treatment with vehicle, while the T/M2_2 community was almost exclusively detected in MRTX1257-treated tissues (fig. S3E). Following this, most of CD8^+^ T cells in the vehicle-treated tumors were found within T/NA and T/M1 communities, while CD8^+^ T cells in the MRTX1257 treatment group were more likely to reside within T/DC, T/M2_1, and T/M2_2 communities (fig. S3F).

Furthermore, the spatial location of the top five communities also varied largely in relation to three assigned tissue domains: normal, interface, and tumor (Fig. 3, B and C). In particular, in the vehicle setting, the T/NA community was situated predominantly in the “normal” nontumor region, and the T/M1 community was restricted to the “interface,” situated as a clear ring around the tumor bulk (fig. S3G). The T/DC community was located just inside the tumor bulk in the vehicle setting but increased in size, and its position moved toward the tumor core following treatment with MRTX1257. The T/M2_1 and T/M2_2 communities were concentrated within the tumor domain and predominantly found within the MRTX1257-treated tumors. Evidently, treatment with MRTX1257 led to a shift in spatial distribution and neighborhood environment of the CD8^+^ T cells.

### Responses of T cell–rich communities to KRAS-G12C inhibition

Communities were defined on the basis of cell types using lineage markers independently of maturation or activation markers. We next sought to explore how cells within the communities responded to KRAS-G12C inhibition based on the maturation and activation markers included in both datasets. Previously, we described how the two subsets of macrophages in this tumor model differed in spatial location and response to treatment with MRTX1257 (*9*). The most notable observation was that the type 2 macrophages increased in cell number and up-regulated activation markers such as programmed cell death ligand 1 (PD-L1) and major histocompatibility complex II. Zooming in on the communities here revealed that the up-regulated expression could be largely attributed to the type 2 macrophages in the T/DC community (fig. S4, A and B).

**Fig. 4.**
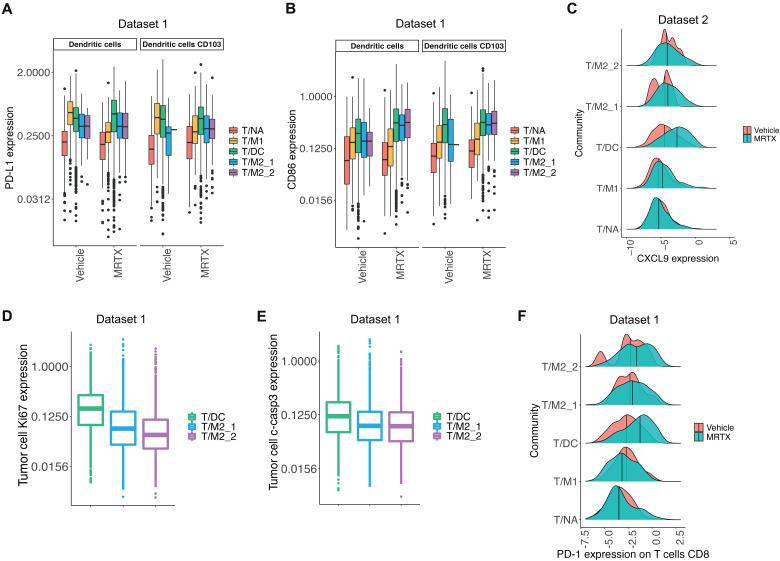
T cell–rich communities respond differently to KRAS-G12C inhibition. (**A** and **B**) Mean expression of (A) PD-L1 and (B) CD86 on DCs and CD103^+^ DCs in T/NA, T/M1, T/DC, T/M2_1, and T/M2_2 communities following vehicle and MRTX1257 treatments for dataset 1 only. Values were log_2_ scaled. (**C**) Mean expression of CXCL9 on DCs, CD103^+^ DCs, macrophages type 1, and macrophages type 2 combined for T/NA, T/M1, T/DC, T/M2_1, and T/M2_2 communities in vehicle- and MRTX1257-treated groups in dataset 2. Values were log_2_ scaled. Center line shows median expression for each treatment group. (**D** and **E**) Mean expression of (D) Ki67 and (E) c-casp3 on tumor cells in T/DC, T/M2_1, and T/M2_2 communities following treatment with MRTX1257 for dataset 1. Values were log_2_ scaled. (**F**) Mean expression of PD-1 on CD8^+^ T cells in communities T/NA, T/M1, T/DC, T/M2_1, and T/M2_2 in vehicle- and MRTX1257-treated groups for dataset 1. Values were log_2_ scaled. Center line shows median expression for each treatment group. MRTX, MRTX1257.

Similarly, we looked at expression of PD-L1 and costimulatory receptor CD86 on DCs. Differences were more noticeable across the communities than between treatment groups, suggesting that DC phenotypes were more influenced by surrounding neighbors than treatment with MRTX1257 (Fig. 4, A and B, and fig. S4C). The behavior of expression varied slightly across these markers, with T/M1 community harboring high PD-L1 but low CD86 expression in the vehicle setting but less so under inhibitor-treated conditions. This high PD-L1 and low CD86 expression is thought to be a characteristic of regulatory or migratory tolerogenic DCs, with a potential to inhibit immune responses (*16*). Alternatively, in T/DC, T/M2_1, and T/M2_2 communities, increased PD-L1 expression on DCs in the MRTX1257 treatment group was accompanied by high CD86 expression, indicative of a more activated DC phenotype, making these cells better equipped to facilitate T cell activation. This differential in activation status was further supported by the expression levels of major histocompatibility complex II, being the highest in the T/DC community (fig. S4D). We previously reported increased expression of T cell chemoattractants chemokine (C-X-C motif) ligand 9 (CXCL9) and CXCL10 in KRAS-G12C inhibitor–treated tumors (*8*). MRTX1257 treatment increased the expression of CXCL9, with the highest overall expression in the T/DC community, suggesting that most of the T cell attraction was occurring within this environment (Fig. 4C). This agrees with our finding that the T/DC community contains the largest T cell density. We also compared proliferative marker Ki67 and apoptotic marker cleaved caspase-3 (c-casp3) expression on tumor cells in the tumor-associated communities (T/DC, T/M2_1, and T/M2_2) and found the highest expression of both markers in the T/DC community (Fig. 4, D and E, and fig. S4, E and F). This demonstrates the high level of activity occurring within this community, where some tumor cells were thriving, while others were dying.

We expected that these different neighborhoods would also have an impact on the phenotype of the T cells, so we investigated their PD-1 expression to understand how T cell activation state changed following KRAS-G12C inhibition, per community. In the T/NA and T/M1 communities, the PD-1 expression was negligible in both treatment groups, compared to the T/DC, T/M2_1, and T/M2_2 communities, where an increase occurred following treatment with MRTX1257, most pronounced in the T/DC community, indicating a switch in cell state from naïve to activated in these communities following KRAS-G12C inhibition (Fig. 4F and fig. S4G). A similar pattern could be seen for CD4^+^ T cells, whereas the T_regs_ had higher PD-1 expression mainly in the T/DC community following MRTX1257 treatment (fig. S4, H and I). However, the expression of lymphocyte-activation gene 3 (LAG-3) protein was also distinctly higher in CD8^+^ T cells, specifically in the tumor-associated communities, in which ~30% of PD-1^+^ T cells were also LAG3^+^ following treatment with MRTX1257. This suggests that activation of the T cells was, in part, accompanied with induction of T cell exhaustion following KRAS-G12C inhibition (fig. S4, J and K).

### Positive and negative regulations of antitumoral immune responses

While the T/DC, T/M2_1, and T/M2_2 communities contained most of the CD8^+^ T cells in the tumor tissue, these cells expressed significant levels of potential exhaustion markers such as PD-1 and LAG-3. Colocalization of PD-L1–expressing macrophages and PD-1^+^ CD8^+^ T cells has recently been highlighted as associated with good response to ICI (*17*, *18*). However, previous attempts to reinvigorate these T cells using MRTX1257 in combination with ICIs anti–PD-1 or anti–PD-L1 and anti-LAG3 had failed to achieve any improved tumor control in our 3LL model (*8*). We therefore wondered whether we could gain any insight into the signals provided to the T cells before moving into the core of the tumor and becoming incapacitated by exhaustion. The high proportion of activated DCs and increased expression of markers associated with T cell attraction and activation following KRAS-G12C inhibition, as well as evidence for local tumor cell death, pointed toward the T/DC community as a potential cytotoxic T cell activation hub.

**Fig. 5.**
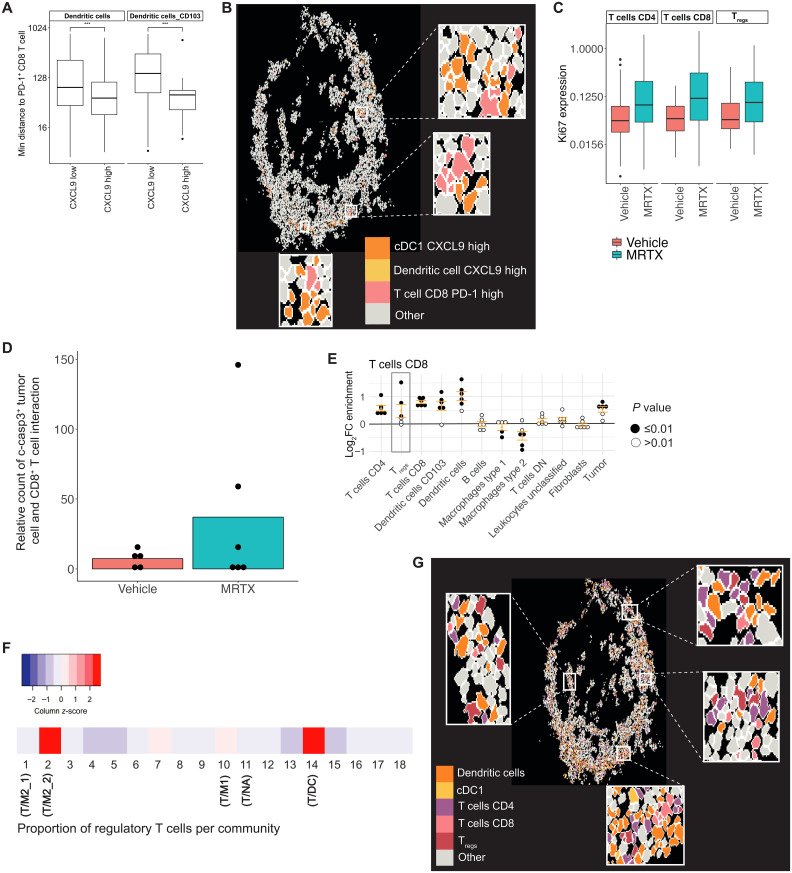
Positive and negative regulations of antitumoral immune responses come together in the T/DC community. (**A**) Minimum distance of DCs and CD103^+^ DCs that have “low” or “high” CXCL9 expression (threshold = 0.5) to PD-1^+^ CD8^+^ T cells within 800 pixels in T/DC community from dataset 2. Distance values were log_2_ scaled. ***P < 0.001. (**B**) Visualization of cell outlines for cells assigned to T/DC community in MRTX1257-treated tissues from dataset 2, with CXCL9-high DCs, CD103^+^ DCs, and PD-1^+^ CD8^+^ T cells colored in to show spatial proximity of these cell phenotypes. Some regions were expanded for easier visualization. (**C**) Mean expression of Ki67 on CD4^+^ and CD8^+^ T cells and T_regs_ within the T/DC community for vehicle and MRTX1257 treatment groups from dataset 2. Values were log_2_ scaled. (**D**) The number of times a c-casp3^+^ tumor cell is found in the 15-pixel neighborhood of a CD8^+^ T cell within the T/DC community, compared across vehicle and MRTX1257 treatment groups for dataset 2, averaged per ROI. Count is relative to the proportion of tumor cells that were c-casp3^+^ in vehicle versus MRTX1257 treatment groups. Each dot represents the value of one ROI. (**E**) Log_2_ fold changes (log_2_FC) in enrichment from neighbouRhood analysis for CD8^+^ T cells in the T/DC community following treatment with MRTX1257. Filled circles represent images from which enrichment was statistically significant compared to randomized spatial arrangements following treatment with MRTX1257 for dataset 2. (**F**) Scaled proportion of T_regs_ contributing to each of the 18 original communities for dataset 2. (**G**) Visualization of cell outlines for cells assigned to the T/DC community in MRTX1257-treated tissues for dataset 2. DCs, CD103^+^ DCs, CD4^+^ and CD8^+^ T cells, and T_regs_ were filled in to illustrate spatial proximity of these cell types. Some regions were expanded for easier visualization. MRTX, MRTX1257.

As noted, CXCL9 expression was the highest within antigen-presenting cells in the T/DC community (Fig. 4C). Separating DCs based on their CXCL9 expression into “CXCL9-low” and “CXCL9-high” groups showed that CXCL9-high DCs had a significantly shorter distance to their nearest PD-1^+^ CD8^+^ T cell, PD-1^+^ CD4^+^ T cells, and PD-1^+^ T_regs_ (Fig. 5A and fig. S5, A and B). Visual inspection indeed confirmed that CXCL9-high DCs were frequently found in proximity to and interacting with PD-1^+^ CD8^+^ T cells (Fig. 5B). CXCL9 expression could therefore be one of the key mediators driving the aggregation of the T cells and DCs within the T/DC community, as part of a mechanism to draw in the activated T cells to launch an antitumor immune response. This would be in agreement with our previous work showing that CXCL9 expression was one of the strongest predictors of ICI (*19*).

We sought to determine further indications of an active antitumoral immune response within this community. A significant increase in Ki67 expression was identified for CD4^+^ and CD8^+^ T cells on MRTX1257 treatment, as well as a similar trend for T_regs_, suggesting that the T cells had increased proliferation following KRAS-G12C inhibition (Fig. 5C). Frequency of casp3^+^ tumor cells was the highest in the T/DC community, and the occurrences in which a c-casp3^+^ tumor cell was found in the 15-pixel neighborhood of a CD8^+^ T cell increased following treatment with MRTX1257 (Fig. 5D). These interactions were primarily found within the T/DC community (fig. S5C). There was no such increase in spatial interactions identified for CD4^+^ or T_regs_ with c-casp3^+^ tumor cells (fig. S5D). These increased interactions point to increased cytotoxicity of the T cells against the tumor cells following KRAS-G12C inhibition, suggesting that the neighborhood of the T/DC community was likely to be able to support the effector function of the CD8^+^ T cells.

We then wondered why such a cytotoxic response was not effective enough to mediate clinical benefit and what could be driving the induction of T cell exhaustion or dysfunction. To identify potential negative regulatory influences of the immune response, we used another way of interrogating spatial relationship by calculating enrichment scores for cells in the near neighborhood, compared to randomized data (*9*, *20*). Following MRTX1257 treatment, it was determined that CD4^+^ T cells and DCs were significantly enriched in the neighborhood of a CD8^+^ T cell within the T/DC community in at least five of the six images, compared to random permutation (Fig. 5E). This is supportive of a microenvironment promoting antitumoral immune response, which was not apparent from T/NA, T/M1, T/M2_1, and T/M2_2 communities (fig. S5, E to H). However, T_regs_ were also significantly enriched in the neighborhood of CD8^+^ T cells in three of the six images, indicating the presence of immune suppressor cells in the vicinity where T cell activation and effector functions may be taking place (Fig. 5E). T_reg_ frequencies were the highest within the T/DC and T/M2_2 communities (Fig. 5F and fig. S5I). This T cell subset also increased in size, most notably in the T/DC and T/M2_2 communities, and, as we saw previously, was showing evidence of increased activation after MRTX1257 treatment (figs. S4I and S5J). T_regs_ were seen intermixed with effector T cells and DCs, suggesting a potential role in locally dampening antitumoral immune responses (Fig. 5G).

### Role for T_regs_ in dampening antitumoral immune responses

**Fig. 6.**
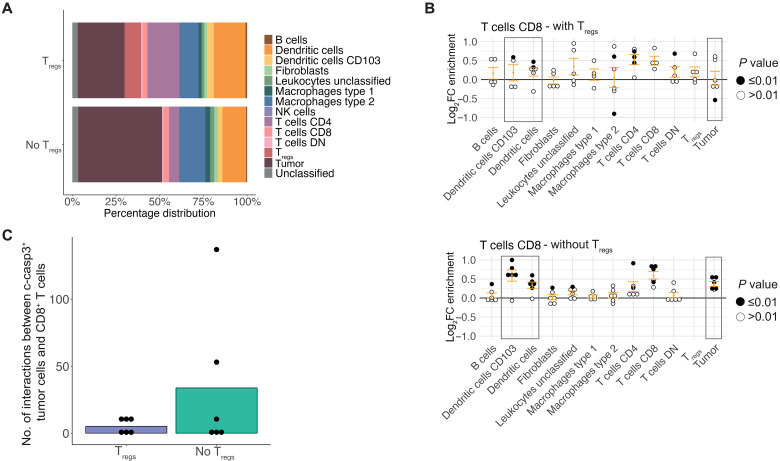
T_regs_ dampen local antitumoral immune responses. (**A**) Percentage distribution of cell types contributing to neighborhoods with T_regs_ (“T_regs_”) and neighborhoods without T_regs_ (“No T_regs_”) within the T/DC community following treatment with MRTX1257. (**B**) Log_2_ fold changes in enrichment from neighbouRhood analysis for CD8^+^ T cells in T_regs_ (top) and no T_regs_ (bottom) neighborhoods within the T/DC community following treatment with MRTX1257. Filled circles represent images from which enrichment value was statistically significant compared to randomization of the spatial arrangements within the T/DC community following treatment with MRTX1257 for dataset 2. (**C**) Number of times a c-casp3^+^ tumor cell is found in the 15-pixel neighborhood of a CD8^+^ T cell within the T/DC community, compared across T_reg_ and no T_reg_ neighborhoods in dataset 2, averaged per ROI. Count is relative to the proportion of tumor cells that were c-casp3^+^ in T_reg_ versus no T_reg_ groups.

Upon revealing the high presence of T_regs_ within the T/DC community and showing that they are enriched within the neighborhood of CD8^+^ T cells and neighboring DCs and CD4^+^ T cells following treatment with MRTX1257, we decided to explore their role within this community in relation to antitumoral immune response. We therefore split up the T/DC community into two neighborhoods: those with presence of T_regs_ or those with absence of T_regs_. The neighborhoods differed slightly in their composition of cell types, with the T_reg_ neighborhood comprising a higher proportion of DCs and CD4^+^ T cells, whereas the no T_reg_ neighborhood contained a higher tumor and type 2 macrophage portion (Fig. 6A).

While the frequency of CD8^+^ T cells within both the T_reg_ and no T_reg_ neighborhoods were similar (fig. S6A), the cellular interactions for CD8^+^ T cells differed substantially between these environments. Despite the slightly higher DC frequency in the T_reg_ neighborhoods, we saw a lack of spatial enrichment between CD8^+^ T cells and both DC subsets when T_regs_ were present, in contrast to a positive spatial enrichment of DCs in the CD8^+^ T cell neighborhood when T_regs_ were absent (Fig. 6B and fig. S6B). In addition, when T_regs_ were not present in the CD8^+^ T cell neighborhood, a strong enrichment of tumor cells was identified following MRTX1257 treatment, compared to slight depletion when the T_regs_ were nearby. Furthermore, the frequency of c-casp3^+^ tumor cells in the CD8^+^ T cell close neighborhood increased on MRTX1257 treatment when T_regs_ were absent (Fig. 6C). We therefore only saw interactions with CD8^+^ T cells indicative of an active antitumoral immune response when the T_regs_ were absent, suggesting an inhibitory role for the T_regs_. These changes in neighborhood enrichment were not observed when comparing CD4^+^ T cells with or without T_regs_ in their neighborhood, indicating that T_reg_ presence may affect CD8^+^, but not CD4^+^, T cell relationships (fig. S6, C and D). However, there were several other changes to the cellular interactions when subsetting the T/DC community based on presence or absence of T_regs_, suggesting that T_regs_ may be affecting the local milieu of this community in many ways, further pointing toward their potential negative influence on antitumoral immune response (fig. S6B). Overall, these analyses indicated that the T/DC community potentially could provide an activating environment for the antitumor cytotoxic T cell response, but the presence of T_regs_ was likely imposing a strong negative influence.

A similar analysis for the T/M2_2 community, also relatively rich in T_regs_ (Fig. 5F), showed that CD8^+^ T cell and T_reg_ interactions focused mainly around fibroblasts, while CD8^+^ T cell interactions with M2 macrophages were largely interrupted in presence of T_regs_ (fig. S6E). Likewise, CD4^+^ T cell interactions with M2 macrophages were also diminished in presence of T_regs_ (fig. S6F). This further solidifies the likely role of T_regs_ suppressing T cell function through down-regulating interactions with antigen-presenting cells following KRAS-G12C inhibition, which occurred across multiple spatial groups.

### T_reg_ spatial communities in human lung NSCLC

While community analysis in mice can help us to identify recurring patterns in a fairly homogeneous and controlled experimental setting, we ultimately aim to translate our findings to better treat patients. Therefore, we wanted to investigate whether communities similar to the mouse communities could be identified in human lung cancer clinical samples, focusing on the communities rich in T_regs_, effector T cells, and DCs, similar to our mouse T/DC community.

**Fig. 7.**
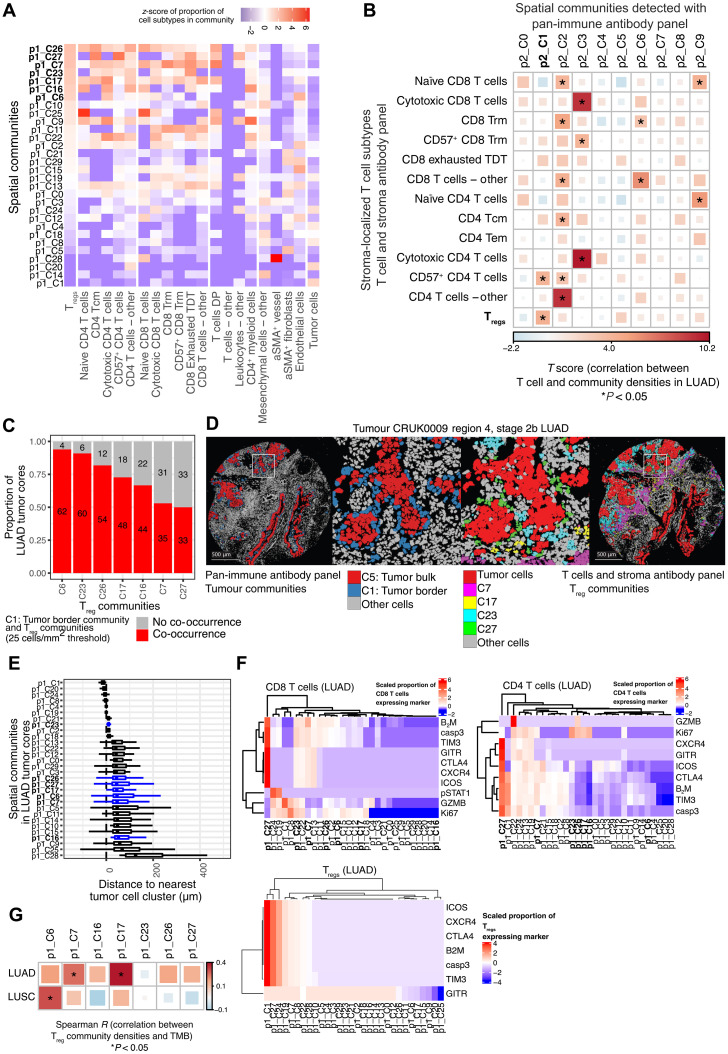
T_reg_ spatial communities are also found in human NSCLC. (**A**) Thirty spatial communities detected in 135 tumor cores from 69 patients with NSCLC. The *z*-score of the proportion of cell subtypes, detected using the T cells and stroma antibody panel (*20*), in each spatial community is shown. Communities p1_C6, p1_C7, p1_C16, p1_C17, p1_C23, p1_C26, and p1_C27 (bold lettering) contain the highest proportions of T_regs_. (**B**) Correlation between the density of stroma-localized T cell subtypes detected using the T cells and stroma antibody panel and the cell density of 10 spatial communities detected using the pan-immune antibody panel, in 66 LUAD tumor cores from 39 patients. Analysis of variance (ANOVA) was conducted using the linear mixed-effects model with patient as a random covariate; *P* values are unadjusted. *P < 0.05. (**C**) Proportion of LUAD tumor cores that contain at least 25 cells/mm^2^ of T_reg_ communities (p1_C6, p1_C7, p1_C16, p1_C17, p1_C23, and p1_C27) and p2_C1: tumor border communities. Sixty-six LUAD tumor cores from 39 patients. (**D**) Pseudo-colored images highlighting cells in the p2_C1: tumor border communities corresponding to cells in T_reg_ communities in serial tumor cores. (**E**) Per-image median distance between cells of a community and their nearest tumor cell cluster. Tumor cell clustering method described in (*71*). Seventy LUAD tumor cores from 40 patients. (**F**) Heatmap displaying the scaled proportion of CD8^+^ T cells, CD4^+^ T cells, and T_regs_ expressing phenotypes of interest. Color scales indicate proportion of cells considered positive, defined by a threshold. β_2_-Microglobulin (β_2_M) is expected to be expressed on all nucleated cells; therefore, this threshold indicates high or low expression (*71*). Seventy LUAD tumor cores from 40 patients. (**G**) Spearman correlation between the density of T_reg_ communities and total harmonized tumor mutational burden. Sixty-nine LUAD cores from 40 patients and 49 LUSC cores from 21 patients. Tcm, central memory T cells; Trm, Tissue resident memory T cells; TDT, Terminally differentiated T cells; DP, double positive; aSMA, alpha smooth muscle actin.

Recently, 151 tumor regions from 81 untreated patients with NSCLC from the TRACERx longitudinal study were analyzed with IMC using two 35-plex antibody panels: A pan-immune cell panel (p2) and a T cells and stroma panel (p1), the latter designed to assess the differentiation states of T cells and stromal cells in greater detail. The T cells and stroma panel provides us with an opportunity to not only match cell type compositions with our mouse data but also to potentially refine the T cell signatures that are associated with them (*14*). Therefore, we chose to detect communities based on the T cells and stroma panel with relatively high granularity by optimizing parameters of the method by Schurch *et al.* (*15*), obtaining 30 communities, including seven that were rich in T_regs_ and other T cells (Fig. 7A). Five of these communities (p1_C7, p1_C17, p1_C23, p1_C26, and p1_C27) were including mature and/or exhausted CD4^+^ and CD8^+^ T cells, similar to the mouse T/DC community. Another characteristic feature of the mouse T/DC community was the presence of DCs, as well as the peritumoral localization. DCs could not be identified from the T cells and stroma panel (p1). However, community analysis of the pan-immune (p2) data had previously identified 10 communities in which “p2_C1: tumor border” also contained DCs and was in the peritumoral region (fig. S7, A to C) (*14*). The presence of p2_C1: tumor border correlated with the density of T_regs_ in LUAD (Fig. 7B), but not in lung squamous cell carcinoma (LUSC) (fig. S7D). In a major subset of patients, we could confirm the co-occurrence of p2_C1: tumor border from the pan-immune panel and T_reg_ communities from the T cells and stroma panel (Fig. 7C) and that, in particular, T_reg_ communities p1_C7, p1_C17, p1_C23, p1_C26, and p1_C27 displayed a peritumoral distribution close to p2_C1: tumor border (Fig. 7, D and E). Homing in on the phenotypes of the T cells associated with these communities revealed that p1_C27 was standing out among the others. CD4^+^ and CD8^+^ T cells in p1_C27 expressed high levels of immune checkpoints (TIM3, GITR, CTLA-4, and ICOS) (Fig. 7F). However, in contrast to the mouse T/DC community, there was no evidence of ongoing T cell activation, with absence of proliferation marker Ki67 and cytotoxicity marker granzyme B (GZMB). The T_regs_ in p1_C27 highly expressed all tested markers and moderate levels of GITR, an immune checkpoint that was recently described to mark the most immune-suppressive T_reg_ subset in NSCLC and associated with PD-1 resistance (*21*). Other T_reg_-rich communities had more moderate expression of immune checkpoints and more frequent expression of Ki67 and GZMB on the CD4^+^ and CD8^+^ T cells, e.g., in p1_C23 and p1_C26. The MYSTIC trial (*22*) demonstrated that the combination of durvalumab (anti–PD-L1) and tremelimumab (anti–CTLA-4) only showed improved survival compared to durvalumab alone in patients with high trimethylboron (TMB) (>20 mutations/Mb). Using the genomics data available for the patients of this TRACERx cohort (*23*), we found that p1_C7 and p1_C17 also correlated with the most increased TMB in LUAD, suggesting that targeting of these T_reg_ communities with anti–CTLA-4 might be beneficial (Fig. 7G). Overall, these data from human lung cancer samples that have not been treated with KRAS inhibitory drugs suggest that cellular communities similar to the mouse T/DC community exist at baseline, where CD4^+^and CD8^+^ T cells, DCs, and T_regs_ were gathered together at the tumor periphery.

### T_regs_ dampen antitumoral immune responses

**Fig. 8.**
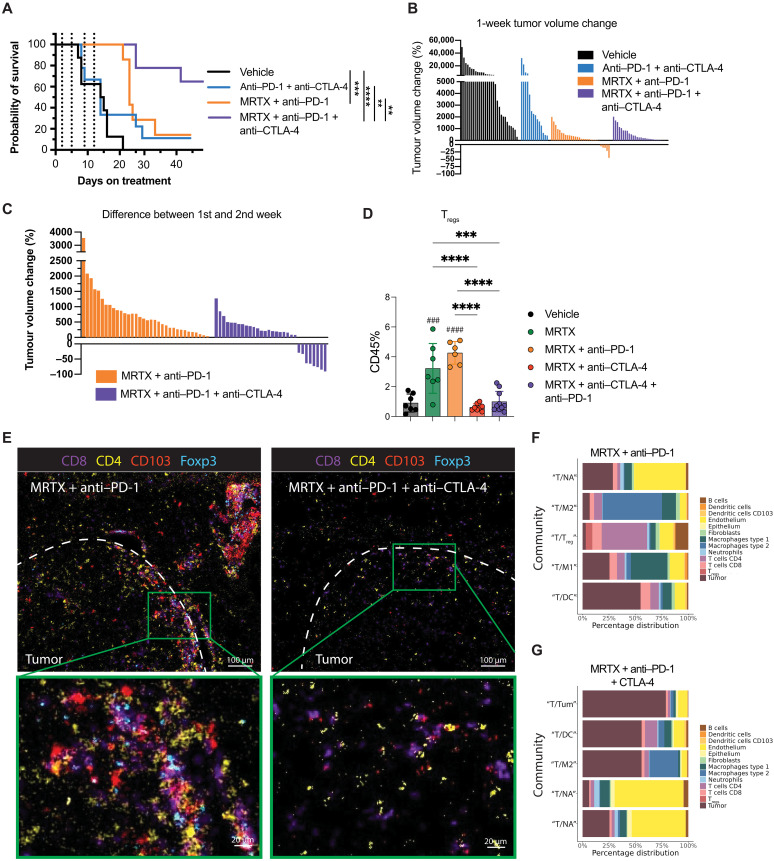
Depletion of T_regs_ rescues antitumoral immune responses. (**A**) Kaplan-Meier analysis of the survival of mice using the 3LL orthotopic lung carcinoma model under vehicle (*n* = 8 mice), anti–PD-1 + anti–CTLA-4 (*n* = 9 mice), MRTX849 + anti–PD-1 (*n* = 7 mice), and MRTX849 + anti–PD-1 + anti–CTLA-4 (*n* = 9 mice) treatment groups. **P < 0.01, ***P < 0.001, ****P < 0.0001. (**B**) Tumor volume changes after 1 week of treatment as measured by microcomputed tomography (μCT) scanning. Multiple tumors per mouse are shown for vehicle (*n* = 8 mice), anti–PD-1 + anti–CTLA-4 (*n* = 9 mice), MRTX849 + anti–PD-1 (*n* = 7 mice), and MRTX849 + anti–PD-1 + anti–CTLA-4 (*n* = 9 mice). (**C**) Tumor volume changes after the second week of treatment as measured by μCT scanning for MRTX849 + anti–PD-1 (*n* = 6 mice) and MRTX849 + anti–PD-1 + anti–CTLA-4 (*n* = 8 mice) treatment groups. (**D**) Percentage of all CD45^+^ cells identified as T_regs_ (gated as CD45^+^ CD3^+^ CD4^+^ Foxp3^+^) measured by flow cytometry in the tumor. Data are mean values ± SD. Each dot represents a mouse. Statistics were calculated using one-way ANOVA. ***P < 0.001, ****P < 0.0001, ^###^*P* < 0.001, ^####^*P* < 0.0001. (**E**) IMC images of a representative tumor area from lungs treated with either MRTX849 + anti–PD-1 or MRTX849 + anti–PD-1 + anti–CTLA-4. Tumor edge is indicated with a dashed line. Underneath a magnification from a CD8 T cell–rich area is shown. For visualization purposes, the images were processed in Fiji with a median and Gaussian filter (radius, 0.5). (**F** and **G**) Top five communities from (F) MRTX849 + anti–PD-1–treated tumors (*n* = 3) and (G) MRTX849 + anti–PD-1 + anti–CTLA-4–treated tumors (*n* = 4). Similarity to communities found in the vehicle- and MRTX849-treated tumors from datasets 1 and 2, based on visual comparison, is indicated with quotation marks around the label (e.g. “T/DC”). Asterisks indicate statistics between samples, and hashtags indicate statistics compared to vehicle. MRTX, MRTX849.

We then went back to the mouse model to investigate whether depleting T_regs_ using an Fc-optimized anti–CTLA-4 antibody (*24*) could promote immune-mediated control of orthotopic 3LL lung tumors treated with KRAS-G12C inhibitor. Anti–CTLA-4 antibody along with anti–PD-1 therapy failed to control tumor growth and extend survival of mice. Similar to what we observed previously, MRTX849 + anti–PD-1 led to short-term tumor control after 1 week on treatment and extended survival, but, eventually, tumors relapsed, and nearly all mice reached their end point within 5 to 6 weeks (Fig. 8, A and B) (*8*). Our previous observations showed that anti–PD-1 antibodies did not improve the response to MRTX849 in this system (*8*). In addition, after 1 week of treatment, combination of MRTX849 + anti–PD-1 with T_reg_-depleting anti–CTLA-4 therapy did not significantly affect tumor growth (Fig. 8B). However, after 2 weeks, tumor volume assessment revealed most tumors from MRTX849 + anti–PD-1 group had relapsed in contrast to triple combination with anti–CTLA-4 where tumors kept regressing (Fig. 8C and fig. S8A). This response pattern continued after 3 weeks of treatment (fig. S8B), and the effect of the triple combination in the long-term resulted in significant tumor control and extension of survival compared to MRTX849 + anti–PD-1, even generating one of the eight completely tumor-free mice, which remained tumor free after withdrawal of treatment for 30 days (Fig. 8A). Variability in tumor control can be observed in the breakdown of tumor growth per mouse (fig. S8C).

Flow cytometry analysis demonstrated increased T_reg_ (CD4^+^Foxp3^+^) infiltration upon MRTX849 treatment, which was exacerbated with the addition of anti–PD-1, while the addition of anti–CTLA-4 therapy induced depletion of T_regs_ in the tumors, as expected (Fig. 8D). We also observed an increased CD8 T cell infiltration with effector and exhausted phenotype (fig. S8, D to F) and elevated CD86 expression on CD103^+^ type 1 DCs (cDC1s), an indication of increased antigen presentation and explainable by reduced transendocytosis in the absence of T_regs_ or Fc receptor–induced activation by the CTLA-4 antibody (fig. S8G).

We generated an additional IMC dataset to investigate the changes in the cellular communities after 7 days of treatment with MRTX845 + anti–PD-1 (*n* = 3 mice, one ROI each) and MRTX849 + anti–PD-1 + anti–CTLA-4 (*n* = 4 mice, one ROI each). Tumors treated with MRTX849 + anti–PD-1 showed a similar architecture to the MRTX849 monotherapy group, except for the increased T_reg_ presence in the local T cell/DC aggregates at the tumor interface (Fig. 8E). This was reflected in the community analysis, where we identified very similar top five CD8 T cell–rich communities as seen in the vehicle and MRTX849 analysis but now with the addition of a community (T/T_reg_) highly enriched in CD4 T cells and T_regs_ (Fig. 8F), as well as in the neighborhood enrichment analysis (fig. S8H). Depletion of T_regs_ led to a disappearance of the densely packed T cell/DC aggregation at the tumor interface (Fig. 8E). This suggests that the T/DC rich community was possibly more of a dysfunctional T cell activation hub, trapping the effector cells while inducing an exhaustive activation state. Instead, in the MRTX849 + anti–PD-1 + anti–CTLA-4 tumors, CD8 T cells were mostly found within the tumor (“T/Tum” and “T/M2”) or in the normal adjacent tissue (“T/NA”) (Fig. 8, E and G), while still interacting with CD4 T cells and CD103^+^ DCs (Fig. 8E and fig. S8H).

In the pulmonary lymph nodes, T_regs_ were not depleted by the addition of anti–CTLA-4 (fig. S8I). This has been observed before and was related to the differential expression of CTLA-4 on T_regs_, being high in tumors and low in the lymph nodes (*25*, *26*), which is in agreement with our data (fig. S8J). Despite this, the lymph nodes showed pronounced T cell activation, evidenced by increased expression of PD-1 and proliferation marker Ki67 in the triple combination treatment group compared to double combinations or single-agent MRTX849 (fig. S8, K and L), again indicative of the priming of a systemic immune response.

## DISCUSSION

Despite objective response rates of roughly 30 to 40%, the licensed KRAS-G12C inhibitors sotorasib (*3*, *4*, *27*) and more recently adagrasib (*28*, *29*) have, so far, achieved only a modest improvement in progression-free survival of about 1 month and no improvement in overall survival. This very limited clinical benefit has been attributed to intrinsic and acquired resistance mechanisms (*30*–*33*), which has led to increased efforts to explore combination therapies. Besides combinatorial targeting of multiple tumor cell intrinsic pathways (*34*–*37*), early preclinical experiments also suggested a potential synergy with immune therapy (*6*, *7*). Durable responses to KRAS-G12C inhibition depended on the presence of intact adaptive immunity (*6*). Furthermore, inhibiting KRAS-G12C was able to turn a cold tumor into a hot proinflammatory environment, potentially supporting antitumor immune responses in combination with ICI (*9*). Similar observations have been made with other KRAS inhibitors and in other model systems, arguing against off-target effects (*6*, *38*–*41*). We found that oncogenic KRAS suppresses interferon signaling within the tumor cells via MYC and down-regulates the antigen presentation machinery and that KRAS-G12C inhibition led to the induction of immunogenic cell death (*8*). Furthermore, oncogenic KRAS signaling mediates the secretion of myeloid cell recruiting and immune polarizing chemokines and cytokines such as chemokine (C-C motif) ligand 2 (CCL2), granulocyte-macrophage colony-stimulating factor, interleukin-10 (IL-10), and transforming growth factor–β, as reviewed in (*42*). Together, there is a multifactorial cascade that supports tissue inflammation after inhibition of KRAS in the tumor cells. However, our own preclinical work demonstrated that despite the presence of TME changes, anti–PD-1 refractory tumors did not become responsive to ICI combinations as a result of KRAS-G12C inhibition (*8*). Similarly, first reports of the safety and efficacy of sotorasib in combination with pembrolizumab or atezolizumab in advanced KRAS-G12C NSCLC have suggested poor response rates in patients that previously progressed on ICI, at least in part due to severe combination toxicities (*11*).

To better understand this persisting resistance to therapy, we further investigated our most immune refractory KRAS-G12C mutant NSCLC model. The 3LL Lewis lung carcinoma model has a history of repeated passaging through immunocompetent mice and, as a result, has developed a complex mixture of immune resistance mechanisms (*43*). In previous work, treating orthotopic 3LL lung tumors with KRAS-G12C inhibitors only provided temporary tumor control, and combinations with anti–PD-1 or anti–PD-L1 and anti-LAG3 failed to give additional benefit (*8*).

Recent studies have demonstrated that the organization and distribution of cellular communities in the tumor can be predictive of clinical outcome (*15*, *44*, *45*), including studies on lung cancer (*13*, *14*, *46*). Similarly, in this study, we investigated the cellular communities in our therapy-resistant 3LL model for evidence of immune-suppressive patterns. Similar to the “suppressed expansion” cluster in breast cancer described by Danenberg *et al.* (*44*), we identified a T cell–rich cellular community (T/DC) with evidence of T cell activation, proliferation, exhaustion, and inhibition. In this cluster, we saw abundant interactions between CD4^+^ and CD8^+^ T cells and mature antigen-presenting DCs, similar to interactions normally seen within the T cell zones of lymph nodes; however, these did not resemble tertiary lymphoid structures, as B cell involvement was sparse. Furthermore, we found evidence of antitumor cytotoxicity within this community, with enrichment for interactions between CD8^+^ PD-1^+^ T cells and c-casp3–expressing tumor cells, suggestive of T cell–induced apoptosis. After treatment with KRAS-G12C inhibitory drug, we observed increased CXCL9 expression, which is involved in T cell recruitment, potentially from the periphery. Tumor-draining lymph nodes (tdLNs) are considered to play a key role in facilitating the antitumor immune response, with the priming of new waves of antitumor T cells by both migratory and resident DCs in tumor and tdLN, which are subsequently attracted to the tumor by chemokine-expressing DC in the TME (*47*). Recent work not only showed that patients responding to ICI harbored more shared T cell receptor clones between tumor and tdLNs but also provided evidence for local expansion and proliferation of CD8^+^ T cells in the TME (*48*). It remains to be determined to what extent the makeup of spatial communities in the TME are a direct reflection of processes occurring in the tdLNs.

While T_regs_ were generally quite scarce in the data, they were found strongly enriched within this T cell/DC–enriched community, and this T_reg_ presence became even more pronounced in the MRTX849 + anti–PD-1 group. T_regs_ have direct and indirect mechanisms to inhibit T cell function. First, T_regs_ can inhibit CD8^+^ T cells directly by secreting or exposing T cell suppressive cytokines, such as IL-10, IL-35, and transforming growth factor–β, by scavenging IL-2, and by the generation of extracellular adenosine (*49*). Unfortunately, we could not investigate these mechanisms within the spatial communities as our panel lacked the markers for these soluble factors.

Second, T_regs_ can compete with CD8^+^ and CD4^+^ T cells for the costimulatory molecules on the antigen-presenting cells, primarily by transendocytosis or trogocytosis of CD80/CD86 through the interaction with CTLA-4 (*50*, *51*). Therefore, blocking of CTLA-4 is able to enhance CD8^+^ T cell responses. CD8/DC interactions were indeed less frequently observed in the neighborhoods containing T_regs_, potentially reducing the opportunity for local activation of CD8^+^ T cells. It was recently shown that depleting T_regs_ with an Fc-modified antibody to promote antibody-dependent cellular cytotoxicity also promotes an inflammatory myeloid response triggered by Fc receptor engagement (*52*). In agreement, depletion of T_regs_ using anti–CTLA-4, in combination with MRTX1257 and anti–PD-1 in our model, led to increased expression of activation markers, including the CTLA-4 ligand CD86 on the DCs.

Notably, the combination of MRTX849, anti–PD-1, and anti–CTLA-4 induced tumor control by enhanced immunity in this otherwise strongly immune-evasive model. Similarly, in another study, we achieved prolonged survival in the same tumor model using a combination of a RAS^G12C^(ON) inhibitor and Src homology region 2 domain-containing phosphatase-2 (SHP-2) inhibition with anti–PD-1 and anti–CTLA-4 (*38*). In addition, Mahadevan *et al.* (*41*) recently showed that KRAS inhibition in a genetic model for pancreatic cancer also led to prolonged survival in a combination therapy with anti–PD-1 and anti–CTLA-4.

Depletion of T_regs_ also led to a disappearance of the densely packed T/DC community at the tumor interface, suggesting that this community played an important role in controlling the cytotoxic activity. This raises the question of whether the presence of a cellular community that is rich in not only activated T cells and DCs but also T_regs_ may predict resistance to the combined therapy of KRAS-G12C inhibition and PD-1 blockade. Spatial investigation of cellular communities in the pretreatment biopsies of the CodeBreak 100/101 clinical trials would allow for correlation with clinical outcome. This is highly relevant as initial results in this study showed that the combination of sotorasib with PD-1 or PD-L1 led to unexpected liver toxicity (*53*); hence, identifying and excluding patients that are resistant to this combination due to T_reg_ activity would help to prevent unnecessary ineffective treatments. Furthermore, this could open up the possibility of using anti–CTLA-4 or other T_reg_ targeting therapies in combination with KRAS-G12C inhibition in selected patients based on a spatial T_reg_ community biomarker.

However, as our data suggest, T_reg_-rich communities are not unique to the KRAS inhibitor–treated conditions, as the T/DC community was present at similar frequencies in the vehicle setting. Likewise, a deeper analysis of the TRACERx IMC cohort (*14*) demonstrated that various T_reg_-rich communities are present in a significant proportion of treatment-naïve patients. Furthermore, Pentimalli *et al.* (*54*) described the presence of a niche rich in cytotoxic T cells, regulatory DCs, and T_regs_, marked by localized expression of CXCL9, similar to our observations, as well as CCL19 and CCL21, in three-dimensional spatial transcriptomics analysis of a patient with high-grade NSCLC. This suggests that DC-, T cell–, and T_reg_-rich communities are a recurring feature in lung cancer and may predict response in a broader context. Several studies in lung cancer so far have indicated frequencies of intratumoral or circulating T_regs_ to be associated with poor outcome (*55*–*57*) or therapy resistance (*58*, *59*), but few have looked into the cellular communities in more detail.

Sorin *et al.* (*13*) identified B cell communities that associated with survival advantage unless they co-occurred with an enrichment of T_regs_. Magen *et al.* (*48*) identified a niche containing progenitor CD8^+^ T cells, dendritic cells enriched in maturation and regulatory molecules (“mregDC”), and CXCL13^+^ T helper cells in ICI responders, while T cell–rich nonresponders were high in T_regs_. CXCL13^+^ T cells were also associated with response to PD-1 blockade in an analysis by Sorin *et al.* (*60*), in contrast to DCs and T_regs_ that were found enriched in another community not significantly correlated to outcome. However, Chen *et al.* (*61*) similarly identified immune hubs associated with response to PD-1 blockade that were rich in stem-like (progenitor-exhausted) T cell factor 7^+^ PD-1^+^ CD8^+^ T cell and mregDCs, also containing T_regs_. This indicates that a careful definition of the T_reg_ communities, including cell states (*21*) and local cytokine environments, will be required to identify accurately predictive biomarkers for resistance to PD-1/PD-L1 blockade. Spatial analysis of pretreatment biopsies from cohorts such as ARCTIC (*62*), MYSTIC (*63*), and CHECKMATE 227 (*64*), comparing anti–PD-L1/PD-1 as monotherapy or in combination with anti–CTLA-4, could help better define the T_reg_ communities that confer resistance to blocking the PD-1/PD-L1 axis alone, while potentially being responsive to the addition of T_reg_ targeting.

Clinical trials for NSCLC that combine nivolumab or durvalumab anti–PD-(L)1 with ipilimumab or tremelimumab anti–CTLA-4 as mentioned above have shown no or only modest additional clinical benefit compared to monotherapy PD-(L)1 blockade (*65*). This may be due to a lack of statistical power resulting from the absence of a biomarker to select the patients that may benefit from additional CTLA-4 blockade. The MYSTIC trial showed that the combination of durvalumab and tremelimumab versus durvalumab monotherapy was performing worse across the whole patient population but showed improved survival in a select patient group with high TMB (*22*). In line with this, we observed two T_reg_ communities with strong similarities to the T_reg_ community identified in mice, which correlated with increased TMB and possibly represent communities that would be more responsive to CTLA-4 blockade or T_reg_ depletion.

Note that it has been heavily debated whether the human anti–CTLA-4 antibodies ipilimumab and tremelimumab are T_reg_ depleting. T_reg_-depleting strategies are being explored clinically, such as an anti–CTLA-4 monoclonal antibody that was Fc engineered to bind FcyRIIIA (botensilimab) (*66*), several different T_reg_-depleting anti-CCR8 antibodies in phase 1/2 studies, mogamulizumab, a CCR4-depleting antibody in phase 1/2 studies (NCT02358473 and NCT02946671) (*67*), and preclinical studies such as the Fc-optimized anti-CD25 (*68*). One of the concerns with anti–CTLA-4 therapies is the enhanced toxicity seen in patients treated with ipilimumab or tremelimumab on top of anti–PD-1 therapy. Local delivery of low-dose anti–CTLA-4 has been proposed to reduce toxicity while maintaining efficacy, such as recently shown for local delivery to the lymphatic basin in melanoma (*69*). However, the site for local delivery of a T_reg_-depleting antibody therapy should be carefully considered. Similar to others before us (*26*, *52*, *68*), we observed how the T_reg_ depletion with anti–CTLA-4 was effective in the tumors, but not in the lymph nodes, most likely due to the differences in CTLA-4 expression. Notably, CTLA-4 levels are increased on activated T_regs_, which allows for selective depletion of these functionally suppressive T_regs_ (*69*), that are elevated both in tumors and in tdLN. Intra- or peritumoral delivery might therefore be a more appropriate approach than systemic anti–CTLA-4 delivery (*47*).

In conclusion, we have used spatial cellular community analysis to investigate the nature of resistance to combined KRAS-G12C and PD-1 inhibition in a strongly immune-evasive mouse model for NSCLC. We identified a cellular community that is rich in T cells and DCs, with evidence of local T cell activation and cytotoxicity but that is inhibited by T_reg_-mediated suppression. Depletion of the T_regs_ led to profound reduction in tumor growth, longer survival, and enhanced, and in some cases sustained, antitumor immunity. Analysis of treatment naïve patients showed that similar communities rich in CD4 and CD8^+^ T cells and T_regs_ were found to co-occur with DCs in the peritumor space and correlated with increased TMB. We propose that a detailed spatial analysis of T_reg_-rich communities in clinical samples from patients treated with KRAS inhibitors, anti–PD-1/PD-L1, or anti–CTLA-4 may provide the foundations for a very specific predictive biomarker.

## MATERIALS AND METHODS

### Study design

The goal of the study was to identify new targets within the spatial cellular communities in the TME of KRAS-G12C inhibitor–treated mouse LUADs to overcome therapy resistance. Previously published data from IMC analysis of Lewis lung carcinoma treated for 7 days with KRAS-G12C inhibitors were reanalyzed to look into the recurring cellular communities that could be potentially linked to therapy resistance. Relationships between cells within the communities were analyzed, looking at cell-cell distances, expression of activation markers, and neighborhood enrichment. To validate the findings, a similar analysis was conducted in a human dataset of treatment naïve NSCLC tumor samples. The identified suppressor cells, namely, T_regs_, were then targeted in vivo using depleting antibodies to verify that these were indeed instrumental in imposing resistance to KRAS-G12C + PD-1 treatment.

### In vivo drug study and IMC

The IMC data of the vehicle and MRTX1257-treated tumors were published previously in van Maldegem *et al.* (*9*) for dataset 1 and Mugarza *et al.* (*8*) for dataset 2. The IMC data of MRTX849 + PD-1– and MRTX849 + PD-1 + CTLA-4–treated tumors were newly generated for this study (dataset 3). For details on samples, staining, imaging, and cell typing, please refer to the above references or the Supplementary Materials.

### Neighbor identification

As described in van Maldegem *et al.* (*9*), cellular neighbors were identified during segmentation using the CellProfiler module “Measure Object Neighbors,” following steps to identify individual cells. Neighbors were identified if a cell boundary was within 15 μm (pixels) of the cell boundary of interest. Neighbor information was obtained for every cell object in each tissue.

### Identification of spatial cellular communities—Mouse data

Each “neighborhood” was identified as a single cell and its local neighbors, defined by 15-μm object relationship output data from segmentation. For each cell, the proportion of each cell type (16 cell types for dataset 1 and 14 cell types for dataset 2) found in its neighborhood was calculated (range, 0 to 1), as demonstrated by the equation below

$$\text{Proportion}=\frac{\text{No}.\text{of cells of type}\;X\;\text{in neighborhood}}{\text{Total}\;\text{no}.\text{of cells in neighborhood}}$$

All cells per dataset were then clustered using Rphenograph, based on the neighbor proportion values calculated. A *k* = 250 clustering input yielded 62 neighborhood communities for dataset 1 and 254 communities for dataset 2.

Dimensionality reduction such as the R implementation of tSNE (*70*) and dendrograms were used to determine which communities were similar and could therefore be analyzed together for dataset 1. Agglomerative clustering using the AgglomerativeClustering function from the sklearn.cluster package in Python 3.9 was then used to group the 62 communities into 18. The clustree package was used to represent the merging of 62 communities into 30 and subsequently 18. Agglomerative clustering was also used to group 254 communities into 18, so that analysis of communities from datasets 1 and 2 were equal and, thus, the datasets could be analyzed in parallel. For validation of the method to identify communities, please refer to the Supplementary Materials.

### Pearson correlation

For comparing cell type relationships per ROI, the proportion of each cell type contributing to each ROI was calculated. For comparing cell type relationships within communities, the proportion of each cell type contributing to each of the 18 communities was calculated. For both instances, this was followed by a Pearson correlation calculation of each cell type pair across all communities. A significant correlation was depicted by a *P* value below 0.05, but significance was tiered as *P < 0.05, **P < 0.01, and ***P < 0.001. Cell types were clustered on the basis of their correlations.

### Neighborhood enrichment analysis

As described in van Maldegem *et al.* (*9*), the neighbouRhood method developed by the Bodenmiller laboratory [https://github.com/BodenmillerGroup/neighbouRhood (*20*)] was used to identify the enrichment of cell types within the 15-pixel neighborhood of each cell compared to random permutations of events, with a modification of only calculating enrichment in the CD8^+^ T cell neighborhood, separated by community and using 1000 rounds of permutation. This was carried out for the community names T/NA, T/M1, T/DC, T/M2_1, and T/M2_2 communities for the MRTX1257 treatment setting.

Neighborhood enrichment analysis was also used for exploring cell pair relationships in the presence or absence of T_regs_ within the T/DC and T/M2_2 communities following MRTX1257 treatment. Enrichment scores were only deemed statistically significant if the *P* value when comparing real neighbors to randomized neighbors was ≤0.01.

### Distance calculations

*X* and *Y* coordinates based on the center of each cell, as generated through segmentation in CellProfiler, were used to calculate distances between cells. The cKDTree function from scipy.spatial package in Python 3.9 was used to compute distances of DCs and CD103^+^ DCs to CD4^+^ and CD8^+^ T cells and T_regs_ that were PD-1^+^ with a distance threshold of 800 pixels within the T/DC community following MRTX1257 treatment.

### Identification of spatial cellular communities—TRACERx data

The TRACERx study (Clinicaltrials.gov no: NCT01888601) is sponsored by University College London (UCL/12/0279) and has been approved by the London–Camden & Kings Cross Research Ethics Committee (13/LO/1546). The community identification method, developed by Schurch *et al.* (*15*), was applied to 139 NSCLC tumor cores from the TRACERx study that were imaged with the pan-immune panel and 135 tumor cores that were imaged with the T cells and stroma panel (*14*) to identify groups of cells that commonly localized near one another.

Briefly, the method is as follows: A window was defined around every cell in an image and its 10 nearest neighboring cells including the center cell. These windows were clustered by their composition with respect to the 18 cell types in the pan-immune panel and the 20 cell types in the T cells and stroma panel (with at least 10 cells on average per image) using MiniBatchKMeans. We optimized the parameters of the method by Schurch *et al.* (*15*) and identified 10 spatial cellular communities from the pan-immune panel and 30 spatial cellular communities using the T cells and stroma panel. Communities were then assigned representative names based on the enrichment of cell densities within them.

Spatial community identities were mapped onto segmented cells and visualized using Cytomapper (*71*), which were then validated by a pathologist’s assessment of serial hematoxylin and eosin–stained tissue sections. The cell density of spatial cellular communities was calculated by taking the number of cells assigned to a spatial cellular community divided by the total tissue area (in cells per square millimeter).

### Association between cell densities of cell subtypes (T cells and stroma panel) and spatial cellular communities (pan-immune panel)—TRACERx data

We correlated the cell density of stroma-localized T cell subtypes detected using the T cells and stroma antibody panel and the cell density of 10 spatial communities detected using the pan-immune antibody panel in cores from the same tumor region. For comparisons with multiple tumor cores/regions per tumor, we used linear mixed-effects model analysis to incorporate patient ID as a random effect. We report the *T* score and *P* value of the model.

### Co-occurrence of spatial cellular communities—TRACERx data

The lowest quartile of the cell densities of T_reg_ communities was approximately 25 cells/mm^2^. Therefore, we used 25 cells/mm^2^ as the threshold to determine whether a spatial community was present or absent in a tumor core. We reported the proportion of paired tumor cores (T cells and stroma panel and pan-immune panel) with at least 25 cells/mm^2^ in p2_C1: tumor border communities and in p1 T_reg_ communities.

### In vivo survival experiment

3LL-ΔNRAS (*37*) was cultured in RPMI 1640 supplemented with 10% fetal bovine serum, 4 mM l-glutamine (Sigma-Aldrich), penicillin (100 U/ml), and streptomycin (100 mg/ml; Sigma-Aldrich). Cell lines were tested for mycoplasma and authenticated by short-tandem repeat DNA profiling by the Francis Crick Institute Cell Services Facility. Cells were allowed to grow for not more than 20 subculture passages.

Intravenous tail vein injections of 10^6^ 3LL-ΔNRAS cells were carried out for orthotopic studies using 8- to 12-week-old male C57BL/6J mice. Mice were euthanized, with an overdose of pentobarbitone, when a humane end point of 15% weight loss was reached or any sign of distress was observed (i.e., hunched, piloerection, and difficulty of breathing). In addition, if a mouse was observed to have a tumor burden in excess of 70% of lung volume when assessed by μCT scanning, they were deemed at risk of rapid deterioration in health and euthanized immediately.

Mice were anesthetized by isoflurane inhalation and scanned using the Quantum GX2 μCT imaging system (PerkinElmer) at a 50-μm isotropic pixel size. Serial lung images were reconstructed and analyzed using Analyze12 (AnalyzeDirect) as previously described in Zaw *et al.* (*72*). Tumor volume changes between time points were calculated as follows: (tumor volume time point 2 − tumor volume time point 1) / tumor volume time point 1 × 100%.

Bristol Myers Squibb antibodies anti–PD-1 (clone 4H2, g1-D265A) and anti–CTLA-4 (clone 9D9, mlgG2a), with mlgG1-D265A and mlgG2a isotype controls, were given twice weekly at 200 μg per dose by intraperitoneal injection. MRTX849 (adagrasib) was given by oral gavage daily at 100 mg/kg for a total of 2 weeks. The mouse work was carried out with approval of the Francis Crick Institute Animal Welfare and Ethical Review Body under UK Home Office Project License P19FC0E42.

### Flow cytometry

Flow cytometry was performed as previously (*8*) using the antibody mixes listed in table S1. Details of staining protocol, data acquisition, and analysis can be found in the Supplementary Materials.

## Supplementary Materials

### This PDF file includes:

Supplementary Materials and Methods Figs. S1 to S8 Table S1 References
