# Supplementary material for: Spatial multiplex analysis of lung cancer reveals that regulatory T cells attenuate KRAS-G12C inhibitor–induced immune responses
Source: Sci Adv. 2024 Nov 1;10(44):eadl6464. doi: 10.1126/sciadv.adl6464 (PMC11529713; doi:10.1126/sciadv.adl6464)
Supplement: Supplementary file 1 — Supplementary Materials and Methods Figs. S1 to S8 Table S1 References [file sciadv.adl6464_sm.pdf]

Science Advances
AAAS

Supplementary Materials for

Spatial multiplex analysis of lung cancer reveals that regulatory T cells attenuate KRAS-G12C inhibitor–induced immune responses

Megan Cole *et al.*

Corresponding author: Julian Downward, julian.downward@crick.ac.uk;
Febe van Maldegem, f.vanmaldegem@amsterdamumc.nl

Sci. Adv. **10**, eadl6464 (2024)
DOI: 10.1126/sciadv.adl6464

This PDF file includes:

Supplementary Text
Figs. S1 to S8
Table S1
References

SUPPLEMENTARY MATERIALS

In vivo drug study and imaging mass cytometry

IMC data was published previously in van Maldegem, et al. (*9*) for dataset 1 and Mugarza, et al.(*8*) for dataset 2 In brief, 10^6 ΔNRAS 3 Lewis lung carcinoma cells (*38*) were injected into the tail vein of 9-11 week old C57BL/6 mice. Following 3 weeks, mice were treated with either 50mg/kg MRTX1257 or Vehicle for 7 consecutive days. Mice were sacrificed on day 8 and lungs were harvested and frozen. Tumors of 3 mice from each treatment group for dataset 1 and 4 mice from each treatment group for dataset 2 were processed for imaging mass cytometry. Tissue slices were stained with a cocktail of antibodies conjugated to heavy metal isotopes and images were obtained using a Hyperion Imaging Mass Cytometer (Standard BioTools). Different antibody panels were used for the generation of datasets 1 and 2 (Supp Figure. 1a, b). In total, 6 images were acquired from both Vehicle and MRTX1257 groups for dataset 1, and the Vehicle group for dataset 2, and 5 images were acquired from the MRTX1257 group for dataset 2. Further information regarding the in vivo study, tissue processing, antibody staining, and image acquisition can be found in van Maldegem, et al., 2021 (*9*).

Image segmentation

Segmentation for the image sets from cohorts 1 and 2 was carried out using CellProfiler v3.1.9, including custom modules by Bodenmiller (https://github.com/BodenmillerGroup/ImcPluginsCP) and Ilastik v1.3.3b1. For cohort 1, a sequential segmentation pipeline was run to identify individual cells from the IMC images. In brief, probability maps were created in Ilastik for the separate identification of lymphocytes, macrophages, fibroblasts, tumor cells and endothelium, while remaining cell objects were identified using a nuclei expansion of 1-pixel. This pipeline also involved domain segmentation through generation of domain probability maps, enabling normal lung tissue, tumor tissue, and the interface region between normal and tumor sections to be identified. See van Maldegem *et al.*(*9*) for further details on the sequential segmentation strategy. For cohort 2, a segmentation pipeline was run which involved a 1-pixel expansion from the cellular nuclei to identify the cell objects. This method did not involve generation of domain information per tissue. For generation of both datasets, segmentation was run using imcyto

(https://github.com/nf-core/imcyto). See 'Data availability' for details of CellProfiler modules used and project files generated for both segmentation methods.

Normalisation, scaling, and clustering

Expression values for each marker were normalised to the mean intensity of Xenon134. Following this, the data for each image across both treatment groups was concatenated, creating a size of 282,837 cells for dataset 1 and 626,070 cells for dataset 2. Each channel was then scaled to the 99th percentile. Mean pixel intensity of 17 cellular markers for dataset 1 (αSMA, B220, CD103, CD11c, CD3, CD44, CD45, CD4, CD68, CD8, EPCAM, F480, LY6G, MHCII, NKp46, PECAM, PVR) and 14 cellular markers for dataset 2 (αSMA, B220, CD103, CD11c, CD3, CD44, CD45, CD4, CD68, CD8, F480, Foxp3, MHC-II and NKp46) were used for clustering using Rphenograph (*73*) with k=20 to identify 30 clusters for dataset 1 and 33 clusters for dataset 2. These clusters formed the basis to identify cell types present in the tissue, including tumor, lymphocytes, myeloid cells, fibroblasts, and endothelium. More details available in van Maldegem *et al*. (*9*) for dataset 1 and Mugarza *et al*. (*8*) for dataset 2.

Validation of method to identify communities

For validation using different input parameters, clustering was run on the neighbourhood proportion information for dataset 1 with a k-input value of 250 to produce 62 communities (as described in 'Community detection' section) and repeated for a k-input value of 350 to yield 47 communities. A tSNE was then used for dimensionality reduction of the cell type proportions contributing to each of the total 109 communities.

For validation across datasets, only the neighbourhood proportion values for the cell types that were shared across datasets 1 and 2 were used, comprising B cells, Dendritic cells, Dendritic cells CD103, Fibroblasts, Macrophages type 1, Macrophages type 2, NK cells, T cells CD4, T cells CD8, T reg cells and Tumor cells. Although both datasets contained cells labelled as Unclassified, they were not included in this analysis due to not being associated with a particular cell phenotype, rather lack of, and therefore could not be directly compared across the two datasets. These neighbourhood proportion values were clustered using Rphenograph with a k-input value of 250, for dataset 1 and 2 separately to yield 18 communities for both dataset 1 and dataset 2 after agglomeration. tSNE was then used for

dimensionality reduction of the cell type proportions contributing to each of the total 36 communities.

Flow cytometry

Mouse lung tumors were cut finely and incubated with digestion solution (collagenase 1 mg/ml; ThermoFisher and DNase I 50 U/ml; Life Technologies) at 37°C for 45 minutes. Cells were then filtered through 70 µm strainers (Falcon) and red blood cells were lysed using ACK buffer (Life Technologies). After washes in PBS, cells were stained with fixable viability dye eFluor870 (BD Horizon) for 30 minutes at 4°C and blocked with CD16/32 antibody (BioLegend) for 10 minutes. Samples were then washed three times in FACS buffer (2 mM EDTA and 0.5% bovine serum albumin in PBS, pH 7.2) before staining of surface markers using fluorescently labelled antibody mixes (See Supplementary Table 1). Cells were fixed with a Fix/lyse solution (eBioscience) after staining. If intracellular staining was carried out, cells were instead fixed with Fix/Perm solution (Invitrogen), followed by intracellular antibody staining. Samples were then resuspended in FACS buffer and analysed using a FACSymphony analyser (BD). Data was analysed using FlowJo.

A

Lymphocytes	
CD45	Pan Leukocyte
CD3	Pan T cell
CD4	T_H Cell
CD8	Cyt Cell
γδTCR	γδT Cell
Foxp3	Regulatory T Cell
B220	B cell
NKp46	NK cell
PD-1	Immune checkpoint

Myeloid cells	
CD45	Pan Leukocyte
Ly6-G	Neutrophils
F4/80	Pan macrophage
CD68	Pan macrophage
CD206	“M2” macrophage
CD11c	DC/macrophage
CD103	cDC1
PD-L1	Immune checkpoint
MHC-II	APC
CD86	Co-stimulation

Tumour cells	
CD44	Stemness
Vimentin	Mesenchymal
C-casp3	Apoptosis
Ki67	Proliferation
pS6	mTOR
PVR	TIGIT receptor

Stroma	
EPCAM	Bronchioles/ epithelium
aSMA	Blood vessels/ fibroblasts
CD31	Endothelium

B

Lymphocytes	
CD45	Pan Leukocyte
CD3	Pan T cell
CD4	T_H Cell
CD8	Cyt Cell
Foxp3	Regulatory T Cell
B220	B cell
NKp46	NK cell
PD-1	Immune checkpoint
LAG3	Exhaustion

Stroma	
aSMA	Blood vessels/ fibroblasts

Myeloid cells	
CD45	Pan Leukocyte
F4/80	Pan macrophage
CD68	Pan macrophage
CD11c	DC/macrophage
CD103	cDC1
PD-L1	Immune checkpoint
MHC-II	APC
CXCL9	T cell attraction

Tumour cells	
CD44	Stemness
MHC-I	Antigen presentation
C-casp3	Apoptosis
Ki67	Proliferation

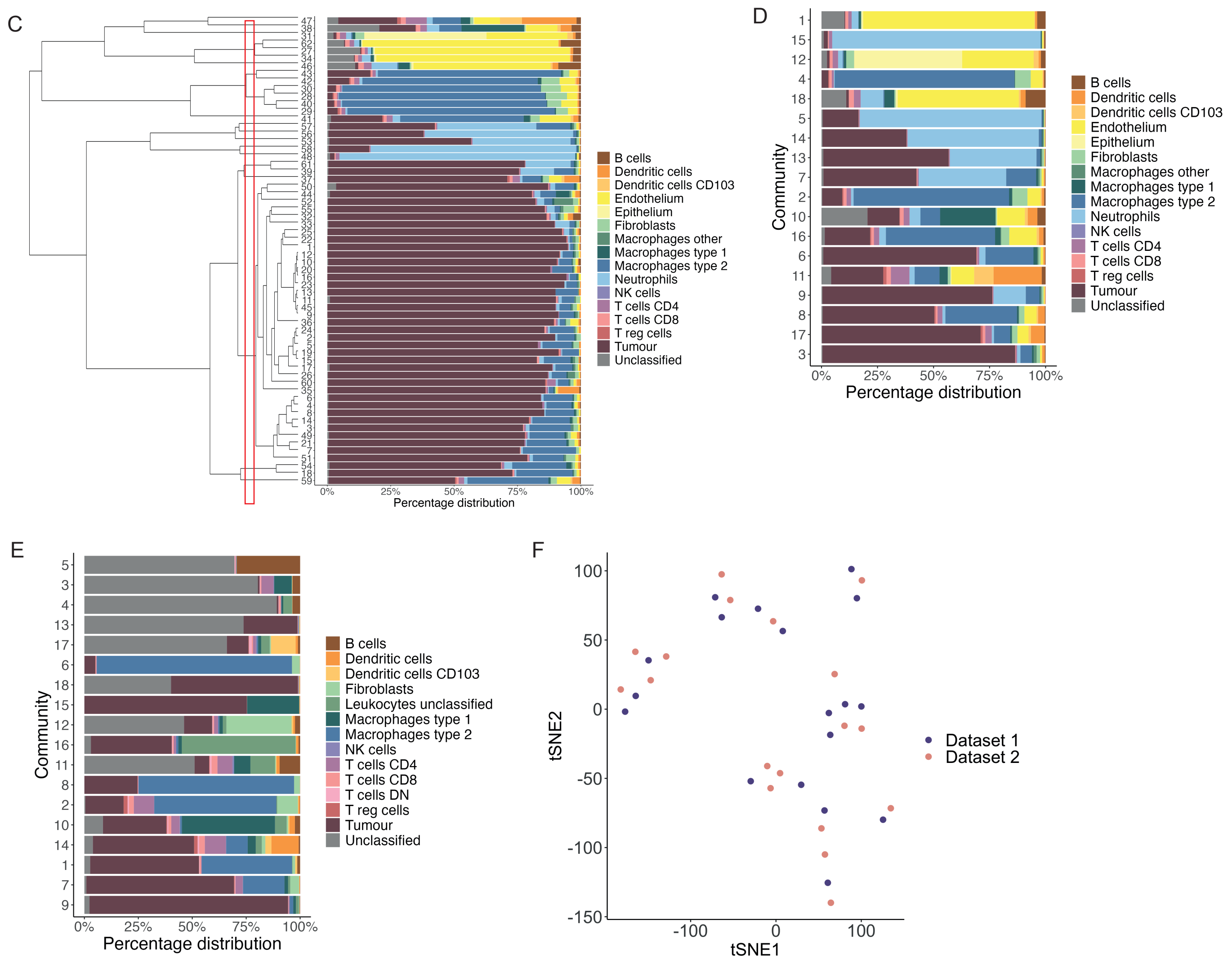

Supplementary Figure 1

Supplementary Figure 1

A second dataset validates the reproducibility of identifying spatial communities.

- **a)** Panel of **a**) 27 antibodies for dataset 1 and **b**) 21 antibodies for dataset 2 that identify multiple cell types across lymphocyte, myeloid, tumor and stromal compartments, as well as immune checkpoint markers and cell phenotypic markers including for detection of proliferation and apoptosis. Additional antibodies for the dataset 2 panel (b) include LAG-3 for identifying exhausted T cells, MHC-I and CXCL9, a chemokine associated with T cell attraction.
- **c)** Percentage distribution of cell types contributing to 62 spatial communities identified from Rphenograph clustering of neighborhood information with k=250. Dendrogram of how 62 communities were agglomerated to 18 communities, with the 18 community point boxed in red.
- **d)** Percentage distribution of all cell types assigned to each of the 18 communities following clustering of neighborhood information for dataset 1.
- **e)** Percentage distribution of all cell types assigned to each of the 18 communities following clustering of neighborhood information for dataset 2.
- **f)** tSNE plot of the 18 communities generated for dataset 1 and 18 communities generated for dataset 2 following Rphenograph clustering based on the proportion of only the cell types shared across both datasets within the neighborhood of each cell.

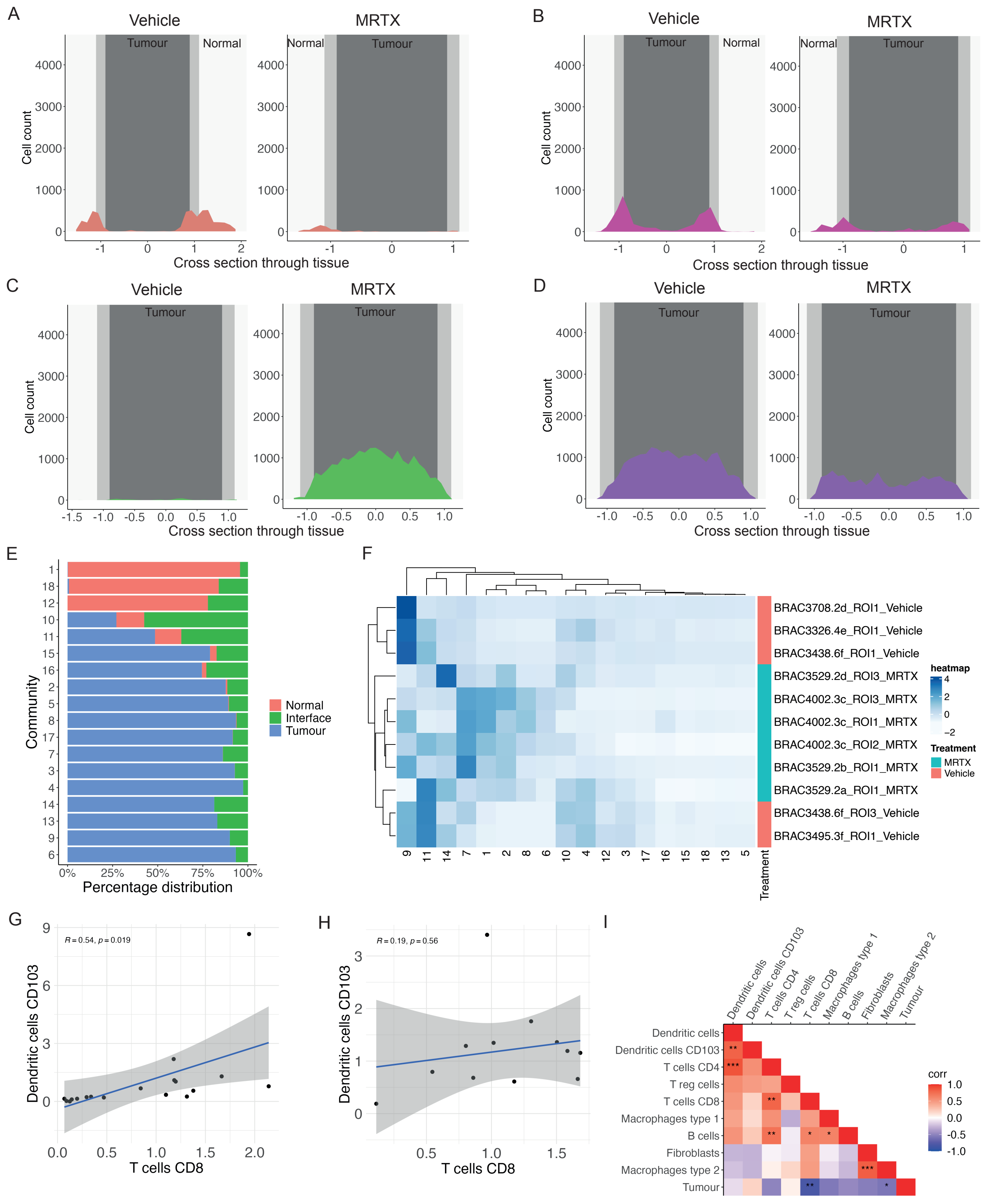

Supplementary Figure 2

Supplementary Figure 2

Identification of spatial communities better captures of tissue organization than cell type alone.

- **a)** Cell count of community relative to cross section through the tissue, where 0 represents the center point of the tumor for community **a**) 18 **b**) 10, **c**) 2 and **d**) 3, for Vehicle (left) and MRTX1257 (right) treatment settings.
- **e)** Percentage distribution of all 18 communities across normal, interface and tumor domains of the tissue for dataset 1.
- **f)** Hierarchical clustering of community proportion per ROI for dataset 2, with use of dendrogram to show relationships between similar ROIs, similar communities, and community distribution across the treatment groups.
- **g)** Pearson correlation calculation of the proportion of Dendritic cells CD103 and T cells $CD8^{+}$in each of the 18 communities for dataset 1.
- **h)** Pearson correlation calculation of the proportion of Dendritic cells CD103 and T cells $CD8^{+}$in each of the 12 ROIs for dataset 1.
- **i)** Pearson correlation calculation on the proportion of each cell type pair within each ROI. * = $p < 0.05$, ** = $p < 0.01$, *** = $p < 0.001$. Cell types clustered based on correlation value.

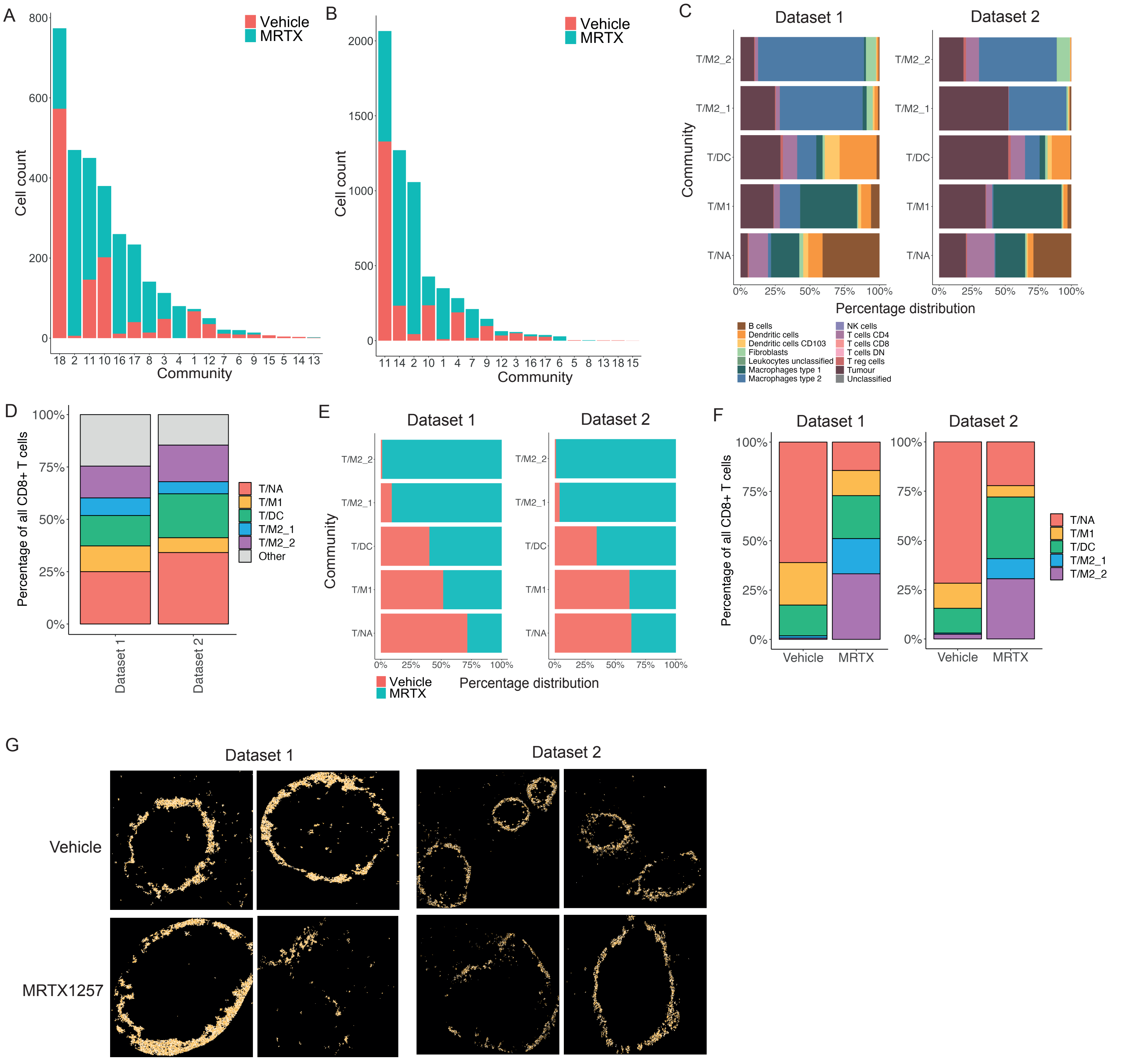

Supplementary Figure 3

Supplementary Figure 3

Both datasets yield similar CD8+ T cell rich communities.

a) Number of $CD8^+$ T cells assigned to each community, with bars colored by distribution of those cells across Vehicle and MRTX1257 treatment groups for **a**) dataset 1 and **b**) dataset 2.

c) Percentage distribution of cell types shared across datasets 1 and 2 contributing to T/NA, T/M1, T/DC, T/M2_1 and T/M2_2 communities.

d) Percentage distribution of all $CD8^+$ T cells in dataset 1 and 2 across T/NA, T/M1, T/DC, T/M2_1 and T/M2_2 communities and all 'other' communities.

e) Distribution of all cells assigned to T/NA, T/M1, T/DC, T/M2_1 and T/M2_2 communities across Vehicle and MRTX1257 treatment groups, relative to the proportion of each treatment group across the whole cohort size for dataset 1 (left) and dataset 2 (right).

f) Percentage of all $CD8^+$ T cells found in the top 5 communities, colored by their distribution across each of the top 5 communities in Vehicle and MRTX1257 treatment groups, for dataset 1 (left) and dataset 2 (right).

g) Visualization of cell outlines for cells assigned to community T/M1, from Vehicle and MRTX1257 treatment groups of datasets 1 (left) and 2 (right).

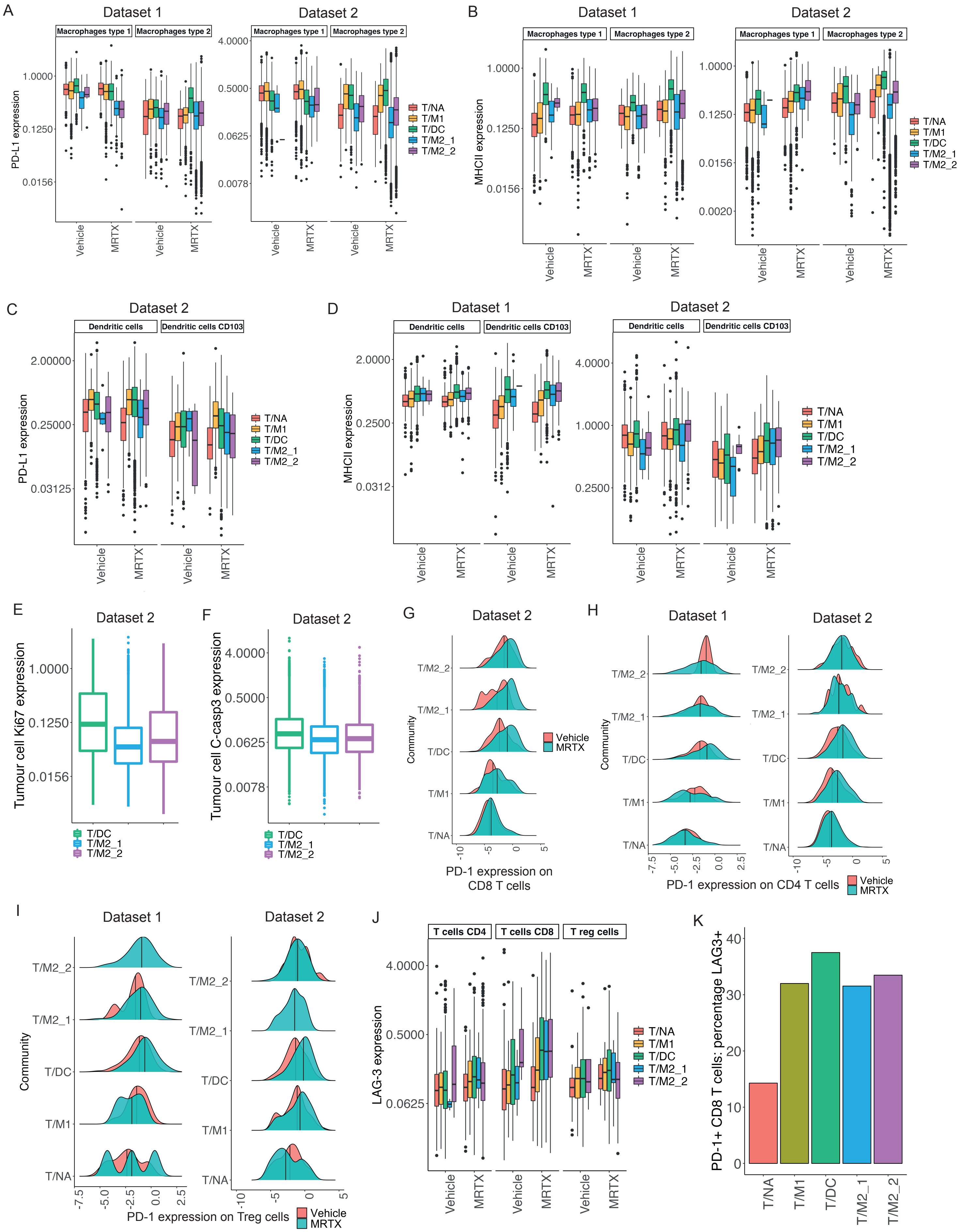

Supplementary Figure 4

Supplementary Figure 4

Spatial communities abundant in CD8+ T cells express different phenotypic markers.

a) Mean expression of **a**) PD-L1 and **b**) MHC-II on macrophages type 1 and type 2 for dataset 1 (left) and dataset 2 (right) in T/NA, T/M1, T/DC, T/M2_1 and T/M2_2 communities for Vehicle and MRTX1257 treatment groups, values were log2 scaled.

c) Mean expression of PD-L1 on dendritic cells and dendritic cells CD103 in communities A-E for Vehicle and MRTX1257 treatment groups, values were log2 scaled.

d) Mean expression of MHC-II on dendritic cells and dendritic cells CD103 for dataset 1 (left) and dataset 2 (right) in T/NA, T/M1, T/DC, T/M2_1 and T/M2_2 communities for Vehicle and MRTX1257 treatment groups, values were log2 scaled.

e) Mean expression of **e**) Ki67 and **f**) cleaved-caspase 3 (c-casp3) on tumor cells in communities C, D and E following treatment with MRTX1257 for dataset 2, values were log2 scaled.

g) Mean expression of PD-1 on **g**) $CD8^+$ T cells for dataset 2, **h**) $CD4^+$ T cells for dataset 1 (left) and dataset 2 (right) and **i**) regulatory T cells for dataset 1 (left) and dataset 2 (right) in T/NA, T/M1, T/DC, T/M2_1 and T/M2_2 communities for Vehicle and MRTX1257-treated groups.

j) Mean expression of LAG-3 on T cells $CD4^+$,T cells $CD8^+$and T reg cells in communities A-E for Vehicle and MRTX1257 treatment groups, values were log2 scaled.

k) Percentage of $PD\text{-}1^+$ $CD8^+$ T cells that are positive for LAG-3 expression (based on a mean expression threshold of 0.5), across T/NA, T/M1, T/DC, T/M2_1 and T/M2_2 communities following MRTX1257treatment.

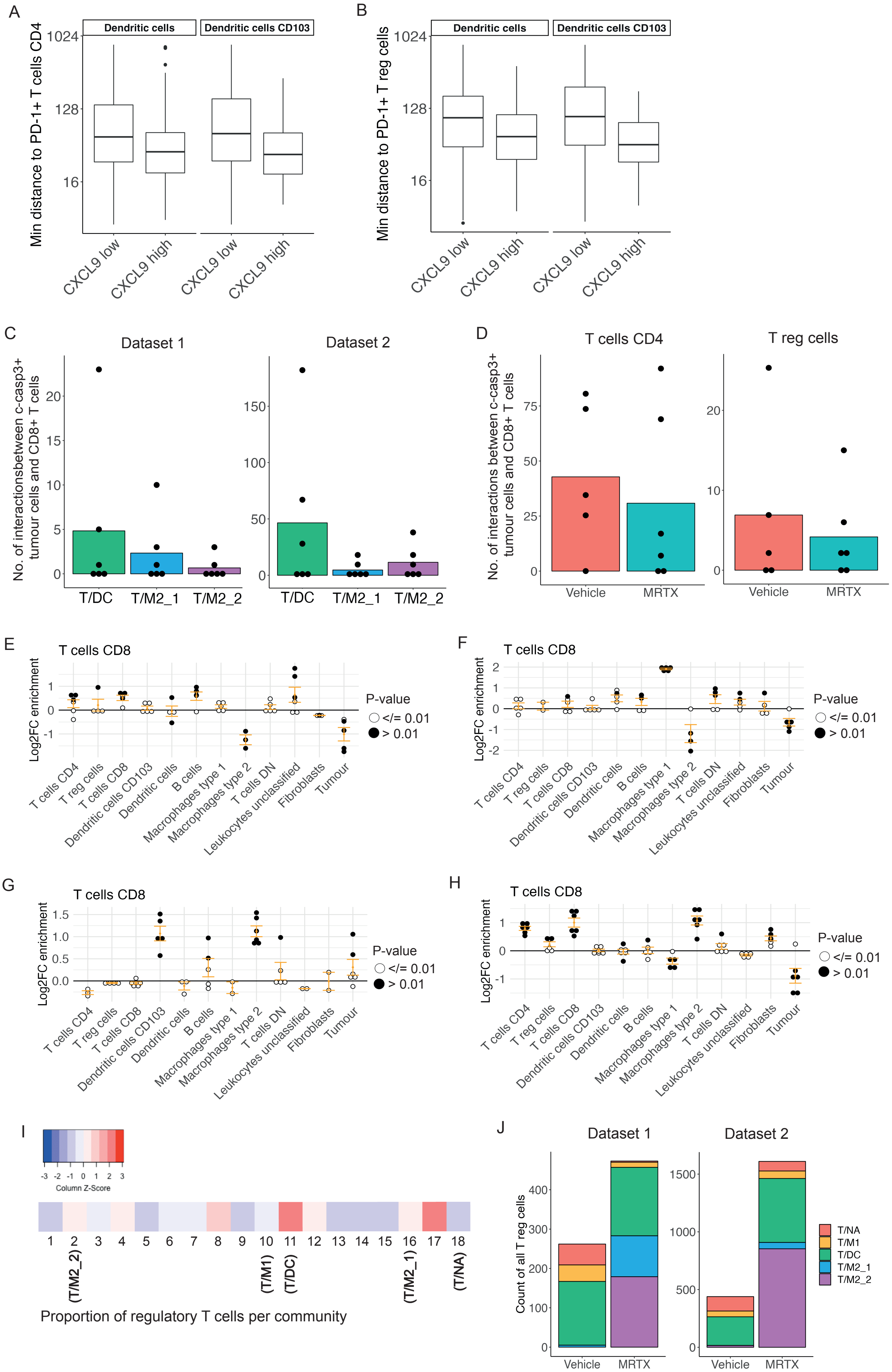

Supplementary Figure 5

Supplementary Figure 5

The T/DC community may host anti-tumoral immune responses.

a) Minimum distance of dendritic cells and CD103^{+} dendritic cells that have 'low' or 'high' CXCL9 expression (based on a threshold of 0.5) to PD-1^{+} CD4^{+} T cells and **b**) regulatory T cells, within 800 pixels in the T/DC community, distance values were log2 scaled.

c) Number of times a cleaved-caspase 3^{+} (c-casp3$^{+)}$ tumor cell is found in the 15-pixel neighborhood of a CD8^{+} T cells within T/DC, T/M2_1 or T/M2_2 communities following MRTX1257 treatment for dataset 1 (left) and dataset 2 (right). Values averaged across ROIs.

d) Number of times a c-casp3^{+} tumor cell is found in the 15-pixel neighborhood of a CD4^{+} T cell (left) and regulatory T cell (right), within the T/DC community, compared across Vehicle and MRTX1257 treatment groups for dataset 2. Count is relative to the proportion of tumor cells that were c-casp3^{+} in Vehicle vs MRTX1257 treatment groups and averaged across ROIs.

e) Log2 fold changes in enrichment from neighbouRhood analysis for CD8^{+} T cells in e) T/NA, f) T/M1, g) T/M2_1 and h) T/M2_2 communities, following treatment with MRTX1257. Filled circles represent images from which enrichment value was statistically significant compared to randomization of the spatial arrangements within all top 5 communities following treatment with MRTX1257.

h) Proportion of regulatory T cells contributing to each of the 18 original communities for dataset 1.

i) Count of regulatory T cells in the top 5 communities, split by Vehicle and MRTX1257 treatment groups for dataset 1 (left) and dataset 2 (right).

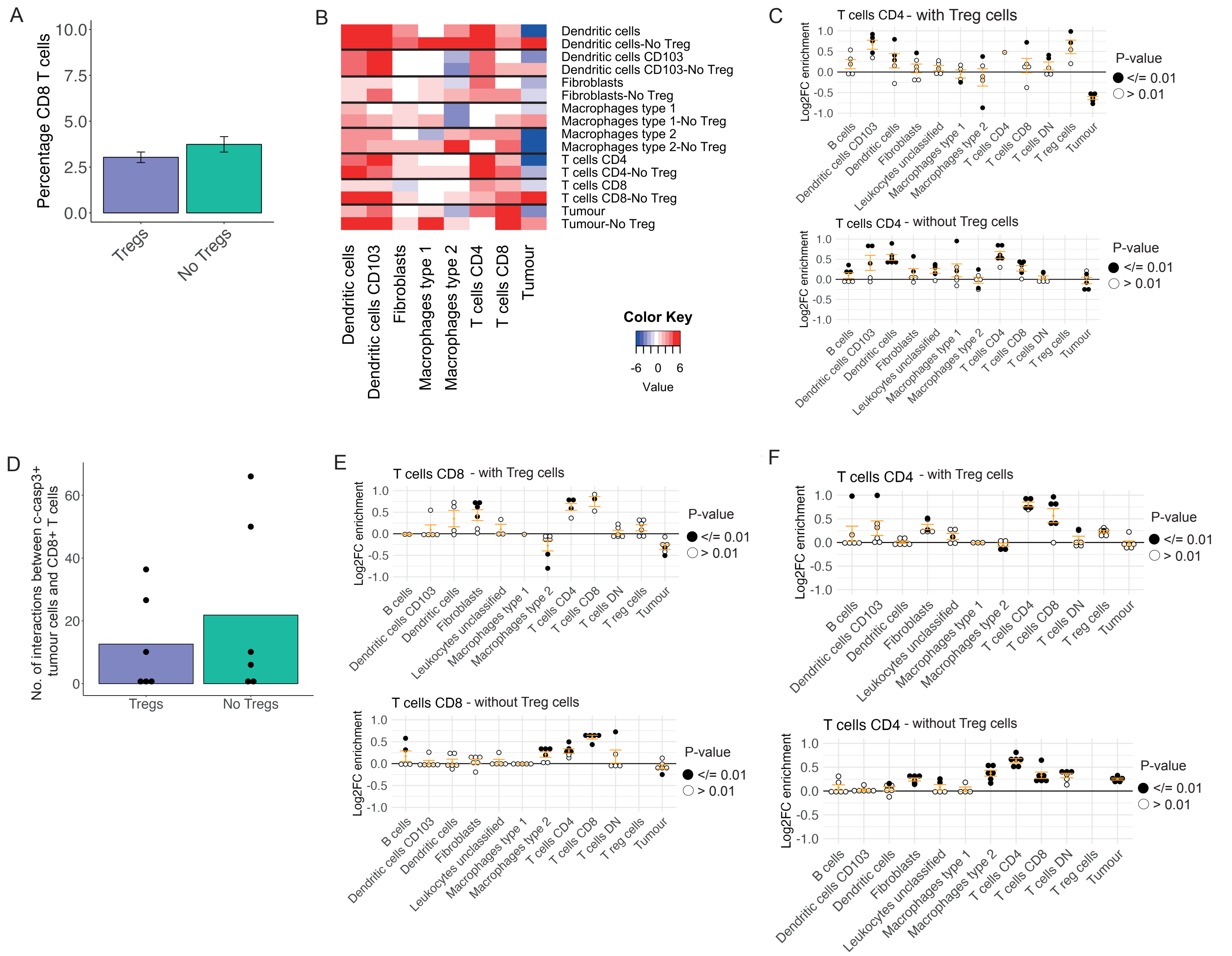

Supplementary Figure 6

Supplementary Figure 6

Treg presence or absence differentiates in local interactions of CD8$^+$ T cells.

a) Percentage of CD8$^+$ T cells within the 'Tregs' and 'No Tregs' neighborhoods of T/DC community in MRTX1257 treatment group, averaged across ROIs.

b) Heatmap of the enrichment scores from neighbouRhood enrichment analysis of each cell type in the neighborhood of every other cell type in the 'Tregs' or 'No Tregs' neighborhood for T/DC community in MRTX1257 treated samples. Score calculated individually per ROI and summed together.

c) Log2 fold changes in enrichment from neighbouRhood analysis for CD8$^+$ T cells in 'Tregs' (top) and 'No Tregs' (bottom) neighborhoods within T/DC community following treatment with MRTX1257. Filled circles represent images from which enrichment value was statistically significant compared to randomization of the spatial arrangements within T/DC community following treatment with MRTX1257 for dataset 2.

d) Number of times a c-casp3$^+$ tumor cell is found in the 15-pixel neighborhood of a CD4$^+$ T cell within T/DC community, compared across 'Tregs' and 'No Tregs' neighborhoods in dataset 2, averaged per ROI following MRTX1257 treatment. Count is relative to the proportion of tumor cells that were c-casp3$^+$ in 'Treg' vs 'No Treg' groups.

e) Log2 fold changes in enrichment from neighbouRhood analysis for **e)** CD8$^+$ T cells and **f**) CD4$^+$ T cells in 'Tregs' (top) and 'No Tregs' (bottom) neighborhoods within T/M2_2 community following treatment with MRTX1257. Filled circles represent images from which enrichment value was statistically significant compared to randomization of the spatial arrangements within the T/M2_2 community following treatment with MRTX1257 for dataset 2.

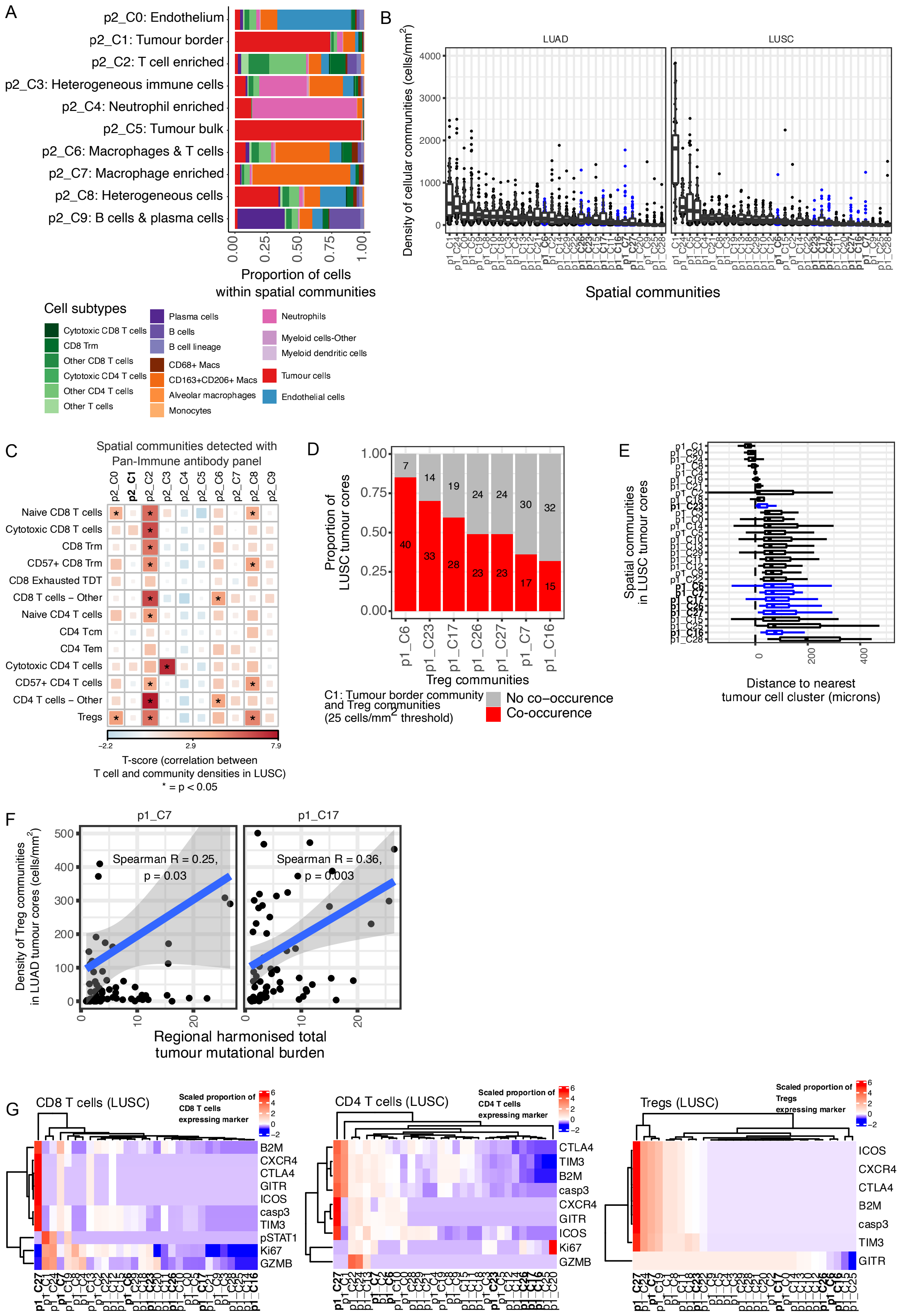

Supplementary Figure 7

Supplementary Figure 7

Seven spatial communities can be identified as Treg rich in human non-small cell lung cancer.

a) Proportion of cell subtypes assigned to each of the ten spatial communities. 139 tumor cores from 68 patients.

b) Density of spatial cellular communities detected in LUAD and LUSC tumor cores. 70 LUAD cores from 40 patients, 51 LUSC cores from 23 patients.

c) Correlation between density of stroma-localized T cell subtypes detected using the T cells & Stroma antibody panel and cell density of ten spatial communities detected using the Pan-immune antibody panel. 47 LUSC tumor cores from 22 patients. LMEM testing the association between T cell density and cell density of communities. Analysis of variance test comparing the LMEM to the null model, *:$p < 0.05$.

d) Proportion of LUSC tumor cores that contain at least 25 cells/mm^2 of Treg communities (*p1_C6, p1_C7, p1_C16, p1_C17, p1_C23, p1_C27*) and *p2_C1: Tumour border* communities. 47 LUSC tumor cores from 22 patients.

e) Per-image median distance between cells of a community and their nearest tumor cell cluster. 51 LUSC tumor cores from 23 patients.

f) Spearman correlation between Treg community density and total harmonized tumor mutational burden. 69 LUAD cores from 40 patients.

g) Scaled proportion of CD8$^+$ T cells, CD4$^+$ T cells and Treg cells expressing phenotypes of interest. Color scales indicate proportion of cells considered positive, defined by a threshold. β2-microglobulin (B2M) is expected to be expressed on all nucleated cells, therefore this threshold indicates high or low expression (72). 51 LUSC tumor cores from 23 patients.

h) Spearman correlation between Treg community density and total harmonized tumor mutational burden. 70 LUAD cores from 41 patients.

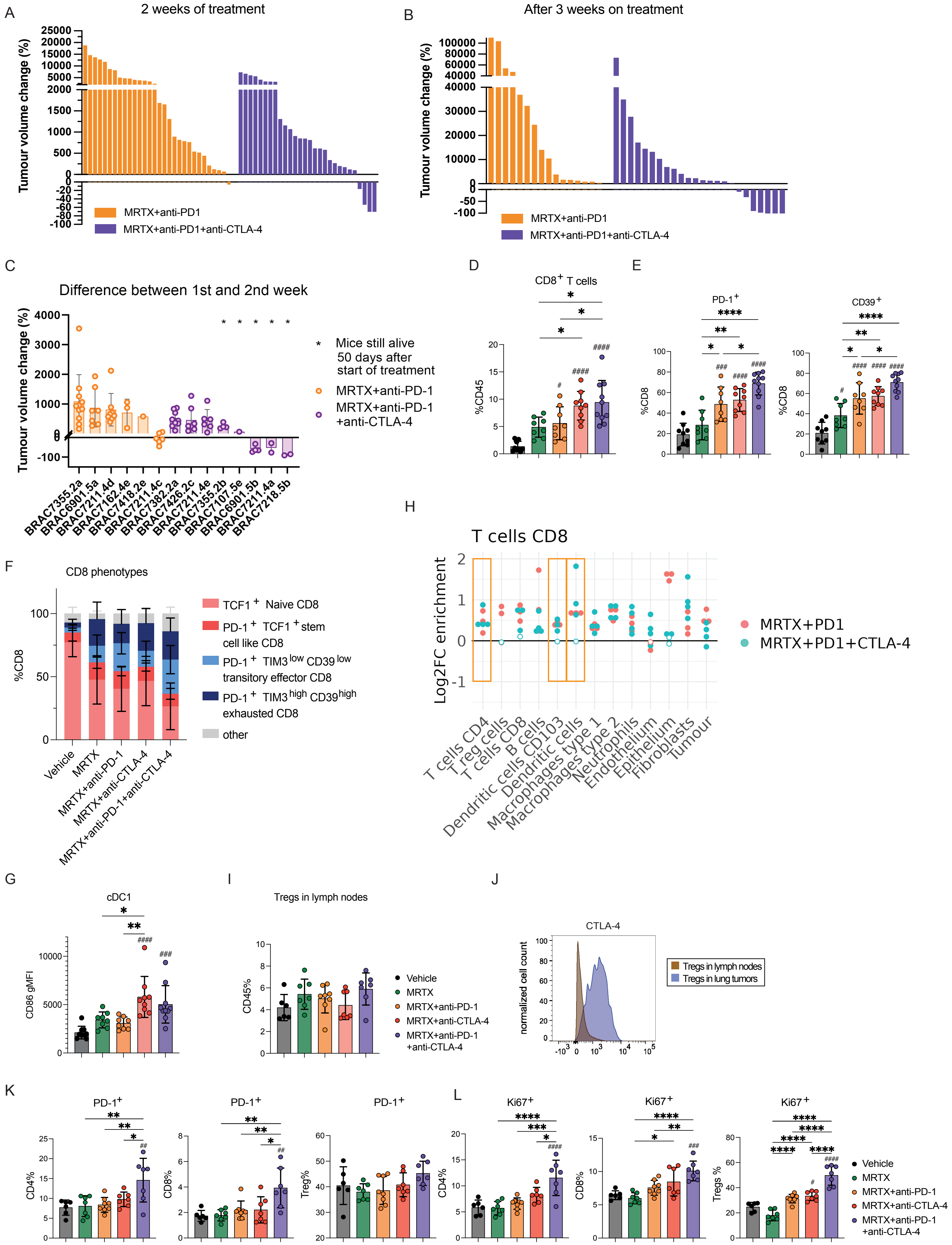

Supplementary Figure 8

Supplementary Figure 8

Changes to the tumor microenvironment in response to Treg depletion.

a) Tumor volume changes after **a)** two weeks and **b)** three weeks of treatment as measured by μCT scanning, for MRTX847+anti-PD-1 and MRTX849+anti-PD-1+anti-CTLA-4 treatment groups.

c) Volume changes between 1st and 2nd week of treatment for individual tumors plotted per mouse. Asterisks indicate point to long-term survivors.

d) Percentage of all CD45$^+$ cells identified as CD8$^+$ T cells (gated as CD45$^+$ CD3$^+$ CD8$^+$) measured by flow cytometry. Data are mean values ± SD. Each dot represents a mouse. Statistics were calculated using one-way ANOVA.

e) Percentage of CD8$^+$ T cells that are PD-1$^+$ and CD39$^+$ measured by flow cytometry in the tumor. Data are mean values ± SD. Each dot represents a mouse. Statistics were calculated using one-way ANOVA.

f) Distribution of CD8$^+$ T cells in the tumor that are TCF1$^+$, PD-1$^+$TCF1-, PD-1$^+$TIM3^{low}CD39^{low} and PD-1$^+$TIM3^{high}CD39^{high} across treatment groups.

g) CD86 expression in cDC1s in the tumor measured by flow cytometry (gMFI; geometric mean fluorescence intensity). Data are mean values ± SD. Each dot represents a mouse. Statistics were calculated using one-way ANOVA.

h) Log2 fold changes in enrichment from neighbouRhood analysis for CD8+ T cells in MRTX+anti-PD-1 and MRTX+anti-PD-1+anti-CTLA-4 treated tumors. Filled circles represent images from which enrichment value was statistically significant compared to randomization of the spatial arrangements within each ROI. Each dot represents one ROI.

i) Percentage of all CD45$^+$ cells identified as regulatory T cells (gated as CD45$^+$ CD3$^+$ CD4$^+$ Foxp3$^+$) measured by flow cytometry in the tumor draining lymph nodes. Data

are mean values ± SD. Each dot represents a mouse. Statistics were calculated using one-way ANOVA.

j) Histogram of CTLA-4 expression on FOXP3$^+$ Tregs following MRTX849 treatment in the tumor (blue) or tumor draining lymph node (brown).

k) Percentage of CD4$^+$ T cells, CD8$^+$ T cells and Treg cells that are PD-1$^+$ and **l)** Ki67$^+$ measured by flow cytometry in the lymph nodes. Data are mean values ± SD. Each dot represents a mouse. Statistics were calculated using one-way ANOVA.

Asterisks: stats between samples, Hashtag, stats compared to vehicle.

Protein	Clone	Fluorophore	Reference	Source
CD3	17A2	FITC	100204	BioLegend
CD45	30-F11	PerCP	103129	BioLegend
Foxp3	FJK-16s	eF660	50-5773-82	eBioscience
CD44	IM7	BV421	103040	BioLegend
CD69	H1.2F3	BV605	104529	BioLegend
CD62L	MEL-14	BV711	104445	BioLegend
PD-1 (CD279)	29F.1A12	BV785	135225	BioLegend
CD8a	53-6.7	BUV395	563786	BD Horizon
CD4	GK1.5	BUV737	612761	BD Horizon
TIM3 (CD366)	RMT3-23	PE	119703	BioLegend
LAG-3 (CD223)	C9B7W	PE-Cy7	125225	BioLegend
CTLA4 (CD152)	UC10-4B9	BV421	106312	BioLegend
TIM3 (CD366)	RMT3-23	BV605	119721	BioLegend
Ki67	B56	BV711	563755	BD Horizon
TCF1/TCF7	C63D9	PE	14456	Cell Signaling
CD39	24DMS1	PE-Cy7	25-0391-80	eBioscience
MHCII (I-A/I-E)	M5/114.15.2	FITC	107605	BioLegend
CD103	2E7	BV421	121421	BioLegend
CD24	M1/69	BV605	101827	BioLegend
CD86	GL-1	BV785	105043	BioLegend
CD11c	HL3	BUV395	564080	BD Horizon
CD11b	M1/70	BUV737	612801	BD Horizon
PD-L1 (CD274)	10F.9G2	PE	124308	BioLegend
FcgRI (CD64)	X54-5/7.1	PE-Cy7	139313	BioLegend

Supplementary Table 1. Antibodies used for flow mass cytometry

REFERENCES AND NOTES

1. H. Borghaei, L. Paz-Ares, L. Horn, D. R. Spigel, M. Steins, N. E. Ready, L. Q. Chow, E. E. Vokes, E. Felip, E. Holgado, F. Barlesi, M. Kohlhäufl, O. Arrieta, M. A. Burgio, J. Fayette, H. Lena, E. Poddubskaya, D. E. Gerber, S. N. Gettinger, C. M. Rudin, N. Rizvi, L. Crinò, G. R. Blumenschein Jr., S. J. Antonia, C. Dorange, C. T. Harbison, F. Graf Finckenstein, J. R. Brahmer, Nivolumab versus docetaxel in advanced nonsquamous non-small-cell lung cancer. *N. Engl. J. Med.* **373**, 1627–1639 (2015).

2. S. N. Gettinger, A. Wurtz, S. B. Goldberg, D. Rimm, K. Schalper, S. Kaech, P. Kavathas, A. Chiang, R. Lilenbaum, D. Zelterman, K. Politi, R. S. Herbst, Clinical features and management of acquired resistance to PD-1 axis inhibitors in 26 patients with advanced non-small cell lung cancer. *J. Thorac. Oncol.* **13**, 831–839 (2018).

3. A. J. de Langen, M. L. Johnson, J. Mazieres, A. C. Dingemans, G. Mountzios, M. Pless, J. Wolf, M. Schuler, H. Lena, F. Skoulidis, Y. Yoneshima, S. W. Kim, H. Linardou, S. Novello, A. J. van der Wekken, Y. Chen, S. Peters, E. Felip, B. J. Solomon, S. S. Ramalingam, C. Dooms, C. R. Lindsay, C. G. Ferreira, N. Blais, C. C. Obiozor, Y. Wang, B. Mehta, T. Varrieur, G. Ngarmchamnanrith, B. Stollenwerk, D. Waterhouse, L. Paz-Ares, K. I. CodeBrea, Sotorasib versus docetaxel for previously treated non-small-cell lung cancer with KRAS(G12C) mutation: A randomised, open-label, phase 3 trial. *Lancet* **401**, 733–746 (2023).

4. F. Skoulidis, B. T. Li, G. K. Dy, T. J. Price, G. S. Falchook, J. Wolf, A. Italiano, M. Schuler, H. Borghaei, F. Barlesi, T. Kato, A. Curioni-Fontecedro, A. Sacher, A. Spira, S. S. Ramalingam, T. Takahashi, B. Besse, A. Anderson, A. Ang, Q. Tran, O. Mather, H. Henary, G. Ngarmchamnanrith, G. Friberg, V. Velcheti, R. Govindan, Sotorasib for lung cancers with *KRAS* p.G12C mutation. *N. Engl. J. Med.* **384**, 2371–2381 (2021).

5. M. Molina-Arcas, A. Samani, J. Downward, Drugging the undruggable: Advances on RAS targeting in cancer. *Genes* **12**, 899 (2021).

6. J. Canon, K. Rex, A. Y. Saiki, C. Mohr, K. Cooke, D. Bagal, K. Gaida, T. Holt, C. G. Knutson, N. Koppada, B. A. Lanman, J. Werner, A. S. Rapaport, T. S. Miguel, R. Ortiz, T. Osgood, J. R. Sun, X. Zhu, J. D. McCarter, L. P. Volak, B. E. Houk, M. G. Fakih, B. H. O'Neil, T. J. Price,

G. S. Falchook, J. Desai, J. Kuo, R. Govindan, D. S. Hong, W. Ouyang, H. Henary, T. Arvedson, V. J. Cee, J. R. Lipford, The clinical KRAS(G12C) inhibitor AMG 510 drives anti-tumour immunity. *Nature* **575**, 217–223 (2019).

7. D. M. Briere, S. Li, A. Calinisan, N. Sudhakar, R. Aranda, L. Hargis, D. H. Peng, J. Deng, L. D. Engstrom, J. Hallin, S. Gatto, J. Fernandez-Banet, A. Pavlicek, K. K. Wong, J. G. Christensen, P. Olson, The $KRAS^{G12C}$ inhibitor MRTX849 reconditions the tumor immune microenvironment and sensitizes tumors to checkpoint inhibitor therapy. *Mol. Cancer Ther.* **20**, 975–985 (2021).

8. E. Mugarza, F. van Maldegem, J. Boumelha, C. Moore, S. Rana, M. Llorian Sopena, P. East, R. Ambler, P. Anastasiou, P. Romero-Clavijo, K. Valand, M. Cole, M. Molina-Arcas, J. Downward, Therapeutic $KRAS^{G12C}$ inhibition drives effective interferon-mediated antitumor immunity in immunogenic lung cancers. *Sci. Adv.* **8**, eabm8780 (2022).

9. F. van Maldegem, K. Valand, M. Cole, H. Patel, M. Angelova, S. Rana, E. Colliver, K. Enfield, N. Bah, G. Kelly, V. S. K. Tsang, E. Mugarza, C. Moore, P. Hobson, D. Levi, M. Molina-Arcas, C. Swanton, J. Downward, Characterisation of tumour microenvironment remodelling following oncogene inhibition in preclinical studies with imaging mass cytometry. *Nat. Commun.* **12**, 5906 (2021).

10. J. Boumelha, S. de Carne Trecesson, E. K. Law, P. Romero-Clavijo, M. A. Coelho, K. W. Ng, E. Mugarza, C. Moore, S. Rana, D. R. Caswell, M. Murillo, D. C. Hancock, P. P. Argyris, W. L. Brown, C. Durfee, L. K. Larson, R. I. Vogel, A. Suarez-Bonnet, S. L. Priestnall, P. East, S. J. Ross, G. Kassiotis, M. Molina-Arcas, C. Swanton, R. Harris, J. Downward, An immunogenic model of KRAS-mutant lung cancer enables evaluation of targeted therapy and immunotherapy combinations. *Cancer Res.* **82**, 3435–3448 (2022).

11. A. Chour, J. Denis, C. Mascaux, M. Zysman, L. Bigay-Game, A. Swalduz, V. Gounant, A. Cortot, M. Darrason, V. Fallet, E. Auclin, C. Basse, C. Tissot, C. Decroisette, P. Bombaron, E. Giroux-Leprieur, L. Odier, S. Brosseau, Q. Creusot, M. Gueçamburu, C. Meersseman, A. Rochand, A. Costantini, C. M. Gaillard, E. Wasielewski, N. Girard, J. Cadranel, C. Lafitte, F. Lebossé, M. Duruisseaux, Brief report: Severe sotorasib-related hepatotoxicity and non-liver

adverse events associated with sequential anti-programmed cell death (ligand) 1 and sotorasib therapy in KRAS^{G12C}-mutant lung cancer. *J. Thorac. Oncol.* **18**, 1408–1415 (2023).

12. C. Giesen, H. A. Wang, D. Schapiro, N. Zivanovic, A. Jacobs, B. Hattendorf, P. J. Schuffler, D. Grolimund, J. M. Buhmann, S. Brandt, Z. Varga, P. J. Wild, D. Gunther, B. Bodenmiller, Highly multiplexed imaging of tumor tissues with subcellular resolution by mass cytometry. *Nat. Methods* **11**, 417–422 (2014).

13. M. Sorin, M. Rezanejad, E. Karimi, B. Fiset, L. Desharnais, L. J. M. Perus, S. Milette, M. W. Yu, S. M. Maritan, S. Dore, E. Pichette, W. Enlow, A. Gagne, Y. Wei, M. Orain, V. S. K. Manem, R. Rayes, P. M. Siegel, S. Camilleri-Broet, P. O. Fiset, P. Desmeules, J. D. Spicer, D. F. Quail, P. Joubert, L. A. Walsh, Single-cell spatial landscapes of the lung tumour immune microenvironment. *Nature* **614**, 548–554 (2023).

14. K. S. S. Enfield, E. Colliver, C. Lee, A. Magness, D. A. Moore, M. Sivakumar, K. Grigoriadis, O. Pich, T. Karasaki, P. S. Hobson, D. Levi, S. Veeriah, C. Puttick, E. L. Nye, M. Green, K. K. Dijkstra, M. Shimato, A. U. Akarca, T. Marafioti, R. Salgado, A. Hackshaw, TRACERx consortium, M. Jamal-Hanjani, F. van Maldegem, N. McGranahan, B. Glass, H. Pulaski, E. Walk, J. L. Reading, S. A. Quezada, C. T. Hiley, J. Downward, E. Sahai, C. Swanton, M. Angelova, Spatial architecture of myeloid and T cells orchestrates immune evasion and clinical outcome in lung cancer. *Cancer Discov.* **14**, 1018–1047 (2024).

15. C. M. Schurch, S. S. Bhate, G. L. Barlow, D. J. Phillips, L. Noti, I. Zlobec, P. Chu, S. Black, J. Demeter, D. R. McIlwain, S. Kinoshita, N. Samusik, Y. Goltsev, G. P. Nolan, Coordinated cellular neighborhoods orchestrate antitumoral immunity at the colorectal cancer invasive front. *Cell* **182**, 1341–1359.e19 (2020).

16. B. Maier, A. M. Leader, S. T. Chen, N. Tung, C. Chang, J. LeBerichel, A. Chudnovskiy, S. Maskey, L. Walker, J. P. Finnigan, M. E. Kirkling, B. Reizis, S. Ghosh, N. R. D'Amore, N. Bhardwaj, C. V. Rothlin, A. Wolf, R. Flores, T. Marron, A. H. Rahman, E. Kenigsberg, B. D. Brown, M. Merad, A conserved dendritic-cell regulatory program limits antitumour immunity. *Nature* **580**, 257–262 (2020).

17. A. Färkkilä, D. C. Gulhan, J. Casado, C. A. Jacobson, H. Nguyen, B. Kochupurakkal, Z. Maliga, C. Yapp, Y. A. Chen, D. Schapiro, Y. Zhou, J. R. Graham, B. J. Dezube, P. Munster, S. Santagata, E. Garcia, S. Rodig, A. Lako, D. Chowdhury, G. I. Shapiro, U. A. Matulonis, P. J. Park, S. Hautaniemi, P. K. Sorger, E. M. Swisher, A. D. D'Andrea, P. A. Konstantinopoulos, Immunogenomic profiling determines responses to combined PARP and PD-1 inhibition in ovarian cancer. *Nat. Commun.* **11**, 1459 (2020).

18. M. Bortolomeazzi, M. R. Keddar, L. Montorsi, A. Acha-Sagredo, L. Benedetti, D. Temelkovski, S. Choi, N. Petrov, K. Todd, P. Wai, J. Kohl, T. Denner, E. Nye, R. Goldstone, S. Ward, G. A. Wilson, M. Al Bakir, C. Swanton, S. John, J. Miles, B. Larijani, V. Kunene, E. Fontana, H. T. Arkenau, P. J. Parker, M. Rodriguez-Justo, K. K. Shiu, J. Spencer, F. D. Ciccarelli, Immunogenomics of colorectal cancer response to checkpoint blockade: Analysis of the KEYNOTE 177 trial and validation cohorts. *Gastroenterology* **161**, 1179–1193 (2021).

19. K. Litchfield, J. L. Reading, C. Puttick, K. Thakkar, C. Abbosh, R. Bentham, T. B. K. Watkins, R. Rosenthal, D. Biswas, A. Rowan, E. Lim, M. Al Bakir, V. Turati, J. A. Guerra-Assuncao, L. Conde, A. J. S. Furness, S. K. Saini, S. R. Hadrup, J. Herrero, S. H. Lee, P. Van Loo, T. Enver, J. Larkin, M. D. Hellmann, S. Turajlic, S. A. Quezada, N. McGranahan, C. Swanton, Meta-analysis of tumor- and T cell-intrinsic mechanisms of sensitization to checkpoint inhibition. *Cell* **184**, 596–614.e14 (2021).

20. D. Schapiro, H. W. Jackson, S. Raghuraman, J. R. Fischer, V. R. T. Zanotelli, D. Schulz, C. Giesen, R. Catena, Z. Varga, B. Bodenmiller, histoCAT: Analysis of cell phenotypes and interactions in multiplex image cytometry data. *Nat. Methods* **14**, 873–876 (2017).

21. A. G. Dykema, J. Zhang, L. S. Cheung, S. Connor, B. Zhang, Z. Zeng, C. M. Cherry, T. Li, J. X. Caushi, M. Nishimoto, A. J. Munoz, Z. Ji, W. Hou, W. Zhan, D. Singh, T. Zhang, R. Rashid, M. Mitchell-Flack, S. Bom, A. Tam, N. Ionta, T. H. K. Aye, Y. Wang, C. A. Sawosik, L. E. Tirado, L. M. Tomasovic, D. VanDyke, J. B. Spangler, V. Anagnostou, S. Yang, J. Spicer, R. Rayes, J. Taube, J. R. Brahmer, P. M. Forde, S. Yegnasubramanian, H. Ji, D. M. Pardoll, K. N. Smith, Lung tumor-infiltrating T_{reg} have divergent transcriptional profiles and function linked to checkpoint blockade response. *Sci. Immunol.* **8**, eadg1487 (2023).

22. N. A. Rizvi, B. C. Cho, N. Reinmuth, K. H. Lee, A. Luft, M. J. Ahn, M. M. van den Heuvel, M. Cobo, D. Vicente, A. Smolin, V. Moiseyenko, S. J. Antonia, S. Le Moulec, G. Robinet, R. Natale, J. Schneider, F. A. Shepherd, S. L. Geater, E. B. Garon, E. S. Kim, S. B. Goldberg, K. Nakagawa, R. Raja, B. W. Higgs, A. M. Boothman, L. Zhao, U. Scheuring, P. K. Stockman, V. K. Chand, S. Peters, MYSTIC Investigators, Durvalumab with or without tremelimumab vs standard chemotherapy in first-line treatment of metastatic non-small cell lung cancer: The MYSTIC phase 3 randomized clinical trial. *JAMA Oncol.* **6**, 661–674 (2020).

23. M. Jamal-Hanjani, G. A. Wilson, N. McGranahan, N. J. Birkbak, T. B. K. Watkins, S. Veeriah, S. Shafi, D. H. Johnson, R. Mitter, R. Rosenthal, M. Salm, S. Horswell, M. Escudero, N. Matthews, A. Rowan, T. Chambers, D. A. Moore, S. Turajlic, H. Xu, S. M. Lee, M. D. Forster, T. Ahmad, C. T. Hiley, C. Abbosh, M. Falzon, E. Borg, T. Marafioti, D. Lawrence, M. Hayward, S. Kolvekar, N. Panagiotopoulos, S. M. Janes, R. Thakrar, A. Ahmed, F. Blackhall, Y. Summers, R. Shah, L. Joseph, A. M. Quinn, P. A. Crosbie, B. Naidu, G. Middleton, G. Langman, S. Trotter, M. Nicolson, H. Remmen, K. Kerr, M. Chetty, L. Gomersall, D. A. Fennell, A. Nakas, S. Rathinam, G. Anand, S. Khan, P. Russell, V. Ezhil, B. Ismail, M. Irvin-Sellers, V. Prakash, J. F. Lester, M. Kornaszewska, R. Attanoos, H. Adams, H. Davies, S. Dentro, P. Taniere, B. O'Sullivan, H. L. Lowe, J. A. Hartley, N. Iles, H. Bell, Y. Ngai, J. A. Shaw, J. Herrero, Z. Szallasi, R. F. Schwarz, A. Stewart, S. A. Quezada, J. Le Quesne, P. Van Loo, C. Dive, A. Hackshaw, C. Swanton, TRACERx Consortium, Tracking the evolution of non-small-cell lung cancer. *N. Engl. J. Med.* **376**, 2109–2121 (2017).

24. M. J. Selby, J. J. Engelhardt, M. Quigley, K. A. Henning, T. Chen, M. Srinivasan, A. J. Korman, Anti-CTLA-4 antibodies of IgG2a isotype enhance antitumor activity through reduction of intratumoral regulatory T cells. *Cancer Immunol. Res.* **1**, 32–42 (2013).

25. F. A. Vargas, A. J. S. Furness, K. Litchfield, K. Joshi, R. Rosenthal, E. Ghorani, I. Solomon, M. H. Lesko, N. Ruef, C. Roddie, J. Y. Henry, L. Spain, A. B. Aissa, A. Georgiou, Y. N. S. Wong, M. Smith, D. Strauss, A. Hayes, D. Nicol, T. O'Brien, L. Martensson, A. Ljungars, I. Teige, B. Frendeus, TRACERx Melanoma, TRACERx Renal, TRACERx Lung consortia, M. Pule, T. Marafioti, M. Gore, J. Larkin, S. Turajlic, C. Swanton, K. S. Peggs, S. A. Quezada,

Fc effector function contributes to the activity of human anti-CTLA-4 antibodies. *Cancer Cell* **33**, 649–663.e4 (2018).

26. A. Marabelle, H. Kohrt, I. Sagiv-Barfi, B. Ajami, R. C. Axtell, G. Zhou, R. Rajapaksa, M. R. Green, J. Torchia, J. Brody, R. Luong, M. D. Rosenblum, L. Steinman, H. I. Levitsky, V. Tse, R. Levy, Depleting tumor-specific Tregs at a single site eradicates disseminated tumors. *J. Clin. Invest.* **123**, 2447–2463 (2013).

27. D. S. Hong, M. G. Fakih, J. H. Strickler, J. Desai, G. A. Durm, G. I. Shapiro, G. S. Falchook, T. J. Price, A. Sacher, C. S. Denlinger, Y. J. Bang, G. K. Dy, J. C. Krauss, Y. Kuboki, J. C. Kuo, A. L. Coveler, K. Park, T. W. Kim, F. Barlesi, P. N. Munster, S. S. Ramalingam, T. F. Burns, F. Meric-Bernstam, H. Henary, J. Ngang, G. Ngarmchamnanrith, J. Kim, B. E. Houk, J. Canon, J. R. Lipford, G. Friberg, P. Lito, R. Govindan, B. T. Li, $KRAS^{G12C}$ inhibition with sotorasib in advanced solid tumors. *N. Engl. J. Med.* **383**, 1207–1217 (2020).

28. P. A. Janne, G. J. Riely, S. M. Gadgeel, R. S. Heist, S. I. Ou, J. M. Pacheco, M. L. Johnson, J. K. Sabari, K. Leventakos, E. Yau, L. Bazhenova, M. V. Negrao, N. A. Pennell, J. Zhang, K. Anderes, H. Der-Torossian, T. Kheoh, K. Velastegui, X. Yan, J. G. Christensen, R. C. Chao, A. I. Spira, Adagrasib in non-small-cell lung cancer harboring a $KRAS^{G12C}$ mutation. *N. Engl. J. Med.* **387**, 120–131 (2022).

29. J. K. Sabari, H. Park, A. W. Tolcher, S. H. I. Ou, E. B. Garon, B. George, P. A. Janne, S. E. Moody, E. Y. Tan, S. K. Sen, D. Peters, X. H. Yan, J. G. Christensen, A. S. Chi, R. S. Heist, KRYSTAL-2: A phase I/II trial of adagrasib (MRTX849) in combination with TNO155 in patients with advanced solid tumors with KRAS G12C mutation. *J. Clin. Oncol.* **39**, TPS146 (2021).

30. S. Suzuki, K. Yonesaka, T. Teramura, T. Takehara, R. Kato, H. Sakai, K. Haratani, J. Tanizaki, H. Kawakami, H. Hayashi, K. Sakai, K. Nishio, K. Nakagawa, KRAS inhibitor resistance in *MET*-Amplified $KRAS^{G12C}$ non-small cell lung cancer induced by RAS- and non-RAS-mediated cell signaling mechanisms. *Clin. Cancer Res.* **27**, 5697–5707 (2021).

31. T. Vaclova, A. Chakraborty, J. Sherwood, S. Ross, D. Carroll, J. C. Barrett, J. Downward, E. C. de Bruin, Concomitant *KRAS* mutations attenuate sensitivity of non-small cell lung cancer cells to KRAS G12C inhibition. *Sci. Rep.* **12**, 2699 (2022).

32. N. Tanaka, J. J. Lin, C. Li, M. B. Ryan, J. Zhang, L. A. Kiedrowski, A. G. Michel, M. U. Syed, K. A. Fella, M. Sakhi, I. Baiev, D. Juric, J. F. Gainor, S. J. Klempner, J. K. Lennerz, G. Siravegna, L. Bar-Peled, A. N. Hata, R. S. Heist, R. B. Corcoran, Clinical acquired resistance to $KRAS^{G12C}$ inhibition through a novel KRAS switch-II pocket mutation and polyclonal alterations converging on RAS-MAPK reactivation. *Cancer Discov.* **11**, 1913–1922 (2021).

33. M. M. Awad, S. Liu, I. I. Rybkin, K. C. Arbour, J. Dilly, V. W. Zhu, M. L. Johnson, R. S. Heist, T. Patil, G. J. Riely, J. O. Jacobson, X. Yang, N. S. Persky, D. E. Root, K. E. Lowder, H. Feng, S. S. Zhang, K. M. Haigis, Y. P. Hung, L. M. Sholl, B. M. Wolpin, J. Wiese, J. Christiansen, J. Lee, A. B. Schrock, L. P. Lim, K. Garg, M. Li, L. D. Engstrom, L. Waters, J. D. Lawson, P. Olson, P. Lito, S. I. Ou, J. G. Christensen, P. A. Janne, A. J. Aguirre, Acquired resistance to $KRAS^{G12C}$ inhibition in cancer. *N. Engl. J. Med.* **384**, 2382–2393 (2021).

34. H. Y. Khan, M. Nagasaka, Y. Li, A. Aboukameel, M. H. Uddin, R. Sexton, S. Bannoura, Y. Mzannar, M. N. Al-Hallak, S. Kim, R. Beydoun, Y. Landesman, H. Mamdani, D. Uprety, P. A. Philip, R. M. Mohammad, A. F. Shields, A. S. Azmi, Inhibitor of the nuclear transport protein XPO1 enhances the anticancer efficacy of KRAS G12C inhibitors in preclinical models of KRAS G12C-mutant cancers. *Cancer Res. Commun.* **2**, 342–352 (2022).

35. S. Khan, J. Wiegand, P. Zhang, W. Hu, D. Thummuri, V. Budamagunta, N. Hua, L. Jin, C. J. Allegra, S. E. Kopetz, M. Zajac-Kaye, F. J. Kaye, G. Zheng, D. Zhou, BCL-X_L PROTAC degrader DT2216 synergizes with sotorasib in preclinical models of $KRAS^{G12C}$-mutated cancers. *J. Hematol. Oncol.* **15**, 23 (2022).

36. S. L. Tammaccaro, P. Prigent, J. C. Le Bail, O. Dos-Santos, L. Dassencourt, M. Eskandar, A. Buzy, O. Venier, J. C. Guillemot, Y. Veeranagouda, M. Didier, E. Spanakis, T. Kanno, M. Cesaroni, S. Mathieu, L. Canard, A. Casse, F. Windenberger, L. Calvet, L. Noblet, S. Sidhu, L. Debussche, J. Moll, I. Valtingojer, TEAD inhibitors sensitize $KRAS^{G12C}$ inhibitors via dual cell cycle arrest in $KRAS^{G12C}$-mutant NSCLC. *Pharmaceuticals* **16**, 553 (2023).

37. M. Molina-Arcas, C. Moore, S. Rana, F. van Maldegem, E. Mugarza, P. Romero-Clavijo, E. Herbert, S. Horswell, L. S. Li, M. R. Janes, D. C. Hancock, J. Downward, Development of combination therapies to maximize the impact of KRAS-G12C inhibitors in lung cancer. *Sci. Transl. Med.* **11**, eaaw7999 (2019).

38. P. Anastasiou, C. Moore, S. Rana, A. de Castro, M. Tomaschko, J. Boumelha, E. Mugarza, C. Blaj, S. de Carné Trécesson, R. Goldstone, J. Smith, E. Quintana, M. Molina-Arcas, J., Downward, Combining RASG12C (ON) inhibitor with SHP2 inhibition sensitises immune excluded lung tumours to immune checkpoint blockade: A strategy for turning cold tumours hot. bioRxiv 2024.01.15.575765 [Preprint] (2024). https://doi.org/10.1101/2024.01.15.575765.

39. S. B. Kemp, N. Cheng, N. Markosyan, R. Sor, I. K. Kim, J. Hallin, J. Shoush, L. Quinones, N. V. Brown, J. B. Bassett, N. Joshi, S. Yuan, M. Smith, W. P. Vostrejs, K. Z. Perez-Vale, B. Kahn, F. Mo, T. R. Donahue, C. G. Radu, C. Clendenin, J. G. Christensen, R. H. Vonderheide, B. Z. Stanger, Efficacy of a small-molecule inhibitor of $Kras^{G12D}$ in immunocompetent models of pancreatic cancer. *Cancer Discov.* **13**, 298–311 (2023).

40. V. Kumarasamy, J. Wang, C. Frangou, Y. Wan, A. Dynka, H. Rosenheck, P. Dey, E. V. Abel, E. S. Knudsen, A. K. Witkiewicz, The extracellular niche and tumor microenvironment enhance KRAS inhibitor efficacy in pancreatic cancer. *Cancer Res.* **84**, 1115–1132 (2024).

41. K. K. Mahadevan, K. M. McAndrews, V. S. LeBleu, S. Yang, H. Lyu, B. Li, A. M. Sockwell, M. L. Kirtley, S. J. Morse, B. A. Moreno Diaz, M. P. Kim, N. Feng, A. M. Lopez, P. A. Guerrero, F. Paradiso, H. Sugimoto, K. A. Arian, H. Ying, Y. Barekatain, L. K. Sthanam, P. J. Kelly, A. Maitra, T. P. Heffernan, R. Kalluri, $KRAS^{G12D}$ inhibition reprograms the microenvironment of early and advanced pancreatic cancer to promote FAS-mediated killing by $CD8^+$ T cells. *Cancer Cell* **41**, 1606–1620.e8 (2023).

42. M. Molina-Arcas, J. Downward, Exploiting the therapeutic implications of KRAS inhibition on tumor immunity. *Cancer Cell* **42**, 338–357 (2024).

43. J. G. Mayo, Biologic characterization of the subcutaneously implanted Lewis lung tumor. *Cancer Chemother. Rep.* **3**, 325–330 (1972).

44. E. Danenberg, H. Bardwell, V. R. T. Zanotelli, E. Provenzano, S. F. Chin, O. M. Rueda, A. Green, E. Rakha, S. Aparicio, I. O. Ellis, B. Bodenmiller, C. Caldas, H. R. Ali, Breast tumor microenvironment structures are associated with genomic features and clinical outcome. *Nat. Genet.* **54**, 660–669 (2022).

45. E. Karimi, M. W. Yu, S. M. Maritan, L. J. M. Perus, M. Rezanejad, M. Sorin, M. Dankner, P. Fallah, S. Dore, D. Zuo, B. Fiset, D. J. Kloosterman, L. Ramsay, Y. Wei, S. Lam, R. Alsajjan, I. R. Watson, G. Roldan Urgoiti, M. Park, D. Brandsma, D. L. Senger, J. A. Chan, L. Akkari, K. Petrecca, M. C. Guiot, P. M. Siegel, D. F. Quail, L. A. Walsh, Single-cell spatial immune landscapes of primary and metastatic brain tumours. *Nature* **614**, 555–563 (2023).

46. D. Tavernari, E. Battistello, E. Dheilly, A. S. Petruzzella, M. Mina, J. Sordet-Dessimoz, S. Peters, T. Krueger, D. Gfeller, N. Riggi, E. Oricchio, I. Letovanec, G. Ciriello, Nongenetic evolution drives lung adenocarcinoma spatial heterogeneity and progression. *Cancer Discov.* **11**, 1490–1507 (2021).

47. K. M. van Pul, M. F. Fransen, R. van de Ven, T. D. de Gruijl, Immunotherapy goes local: The central role of lymph nodes in driving tumor infiltration and efficacy. *Front. Immunol.* **12**, 643291 (2021).

48. A. Magen, P. Hamon, N. Fiaschi, B. Y. Soong, M. D. Park, R. Mattiuz, E. Humblin, L. Troncoso, D. D'Souza, T. Dawson, J. Kim, S. Hamel, M. Buckup, C. Chang, A. Tabachnikova, H. Schwartz, N. Malissen, Y. Lavin, A. Soares-Schanoski, B. Giotti, S. Hegde, G. Ioannou, E. Gonzalez-Kozlova, C. Hennequin, J. Le Berichel, Z. Zhao, S. C. Ward, I. Fiel, B. Kou, M. Dobosz, L. Li, C. Adler, M. Ni, Y. Wei, W. Wang, G. S. Atwal, K. Kundu, K. J. Cygan, A. M. Tsankov, A. Rahman, C. Price, N. Fernandez, J. He, N. T. Gupta, S. Kim-Schulze, S. Gnjatic, E. Kenigsberg, R. P. Deering, M. Schwartz, T. U. Marron, G. Thurston, A. O. Kamphorst, M. Merad, Intratumoral dendritic cell-CD4^{+} T helper cell niches enable CD8^{+} T cell differentiation following PD-1 blockade in hepatocellular carcinoma. *Nat. Med.* **29**, 1389–1399 (2023).

49. A. Schmidt, N. Oberle, P. H. Krammer, Molecular mechanisms of treg-mediated T cell suppression. *Front. Immunol.* **3**, 51 (2012).

50. M. Tekguc, J. B. Wing, M. Osaki, J. Long, S. Sakaguchi, Treg-expressed CTLA-4 depletes CD80/CD86 by trogocytosis, releasing free PD-L1 on antigen-presenting cells. *Proc. Natl. Acad. Sci. U.S.A.* **118**, e2023739118 (2021).

51. A. Kennedy, E. Waters, B. Rowshanravan, C. Hinze, C. Williams, D. Janman, T. A. Fox, C. Booth, A. M. Pesenacker, N. Halliday, B. Soskic, S. Kaur, O. S. Qureshi, E. C. Morris, S. Ikemizu, C. Paluch, J. Huo, S. J. Davis, E. Boucrot, L. S. K. Walker, D. M. Sansom, Differences in CD80 and CD86 transendocytosis reveal CD86 as a key target for CTLA-4 immune regulation. *Nat. Immunol.* **23**, 1365–1378 (2022).

52. I. Yofe, T. Landsberger, A. Yalin, I. Solomon, C. Costoya, D. F. Demane, M. Shah, E. David, C. Borenstein, O. Barboy, I. Matos, K. S. Peggs, S. A. Quezada, I. Amit, Anti-CTLA-4 antibodies drive myeloid activation and reprogram the tumor microenvironment through FcγR engagement and type I interferon signaling. *Nat. Cancer* **3**, 1336–1350 (2022).

53. B. T. Li, G. S. Falchook, G. A. Durm, T. F. Burns, F. Skoulidis, S. S. Ramalingam, A. Spira, C. M. Bestvina, S. B. Goldberg, R. Veluswamy, W. T. Iams, A. A. Chiappori, C. R. Lemech, A. R. Meloni, V. Ebiana, T. Dai, D. M. Gauto, T. L. Varrieur, W. J. Snyder, R. Govindan, OA03.06 CodeBreaK 100/101: First report of safety/efficacy of sotorasib in combination with pembrolizumab or atezolizumab in advanced KRAS p.G12C NSCLC. *J. Thorac. Oncol.* **17**, S10–S11 (2022).

54. T. M. Pentimalli, S. Schallenberg, D. León-Periñán, I. Legnini, I. Theurillat, G. Thomas, A. Boltengagen, S. Fritzsche, J. Nimo, L. Ruff, G. Dernbach, P. Jurmeister, S. Murphy, M. T. Gregory, Y. Liang, M. Cordenonsi, S. Piccolo, F. Coscia, A. Woehler, N. Karaiskos, F. Klauschen, N. Rajewsky, High-resolution molecular atlas of a lung tumor in 3D. bioRxiv 2023.05.10.539644 [Preprint] (2024). https://doi.org/10.1101/2023.05.10.539644.

55. K. Shimizu, M. Nakata, Y. Hirami, T. Yukawa, A. Maeda, K. Tanemoto, Tumor-infiltrating Foxp3+ regulatory T cells are correlated with cyclooxygenase-2 expression and are associated with recurrence in resected non-small cell lung cancer. *J. Thorac. Oncol.* **5**, 585–590 (2010).

56. D. H. Kang, C. Chung, P. Sun, D. H. Lee, S. I. Lee, D. Park, J. S. Koh, Y. Kim, H. S. Yi, J. E. Lee, Circulating regulatory T cells predict efficacy and atypical responses in lung cancer patients treated with PD-1/PD-L1 inhibitors. *Cancer Immunol. Immunother.* **71**, 579–588 (2022).

57. R. Saleh, E. Elkord, $FoxP3^+$ T regulatory cells in cancer: Prognostic biomarkers and therapeutic targets. *Cancer Lett.* **490**, 174–185 (2020).

58. M. van Gulijk, A. van Krimpen, S. Schetters, M. Eterman, M. van Elsas, J. Mankor, L. Klaase, M. de Bruijn, M. van Nimwegen, T. van Tienhoven, W. van Ijcken, L. Boon, J. van der Schoot, M. Verdoes, F. Scheeren, S. H. van der Burg, B. N. Lambrecht, R. Stadhouders, F. Dammeijer, J. Aerts, T. van Hall, PD-L1 checkpoint blockade promotes regulatory T cell activity that underlies therapy resistance. *Sci. Immunol.* **8**, eabn6173 (2023).

59. L. van Hooren, S. M. Handgraaf, D. J. Kloosterman, E. Karimi, L. van Mil, A. A. Gassama, B. G. Solsona, M. H. P. de Groot, D. Brandsma, D. F. Quail, L. A. Walsh, G. R. Borst, L. Akkari, $CD103^+$ regulatory T cells underlie resistance to radio-immunotherapy and impair $CD8^+$ T cell activation in glioblastoma. *Nat. Cancer* **4**, 665–681 (2023).

60. M. Sorin, E. Karimi, M. Rezanejad, M. W. Yu, L. Desharnais, S. A. C. McDowell, S. Dore, A. Arabzadeh, V. Breton, B. Fiset, Y. Wei, R. Rayes, M. Orain, F. Coulombe, V. S. K. Manem, A. Gagne, D. F. Quail, P. Joubert, J. D. Spicer, L. A. Walsh, Single-cell spatial landscape of immunotherapy response reveals mechanisms of CXCL13 enhanced antitumor immunity. *J. Immunother. Cancer* **11**, e005545 (2023).

61. J. H. Chen, L. T. Nieman, M. Spurrell, V. Jorgji, L. Elmelech, P. Richieri, K. H. Xu, R. Madhu, M. Parikh, I. Zamora, A. Mehta, C. S. Nabel, S. S. Freeman, J. D. Pirl, C. Lu, C. B. Meador, J. L. Barth, M. Sakhi, A. L. Tang, S. Sarkizova, C. Price, N. F. Fernandez, G. Emanuel, J. He, K. Van Raay, J. W. Reeves, K. Yizhak, M. Hofree, A. Shih, M. Sade-Feldman, G. M. Boland, K. Pelka, M. J. Aryee, M. Mino-Kenudson, J. F. Gainor, I. Korsunsky, N. Hacohen, Human lung cancer harbors spatially organized stem-immunity hubs associated with response to immunotherapy. *Nat. Immunol.* **25**, 644–658 (2024).

62. D. Planchard, N. Reinmuth, S. Orlov, J. R. Fischer, S. Sugawara, S. Mandziuk, D. Marquez-Medina, S. Novello, Y. Takeda, R. Soo, K. Park, M. McCleod, S. L. Geater, M. Powell, R. May, U. Scheuring, P. Stockman, D. Kowalski, ARCTIC: Durvalumab with or without tremelimumab as third-line or later treatment of metastatic non-small-cell lung cancer. *Ann. Oncol.* **31**, 609–618 (2020).

63. E. B. Garon, B. C. Cho, N. Reinmuth, K. H. Lee, A. Luft, M. J. Ahn, G. Robinet, S. Le Moulec, R. Natale, J. Schneider, F. A. Shepherd, M. C. Garassino, S. L. Geater, Z. P. Szekely, T. Van Ngoc, F. Liu, U. Scheuring, N. Patel, S. Peters, N. A. Rizvi, Patient-reported outcomes with durvalumab with or without tremelimumab versus standard chemotherapy as first-line treatment of metastatic non-small-cell lung cancer (MYSTIC). *Clin. Lung Cancer* **22**, 301–312.e308 (2021).

64. M. D. Hellmann, L. Paz-Ares, R. Bernabe Caro, B. Zurawski, S. W. Kim, E. Carcereny Costa, K. Park, A. Alexandru, L. Lupinacci, E. de la Mora Jimenez, H. Sakai, I. Albert, A. Vergnenegre, S. Peters, K. Syrigos, F. Barlesi, M. Reck, H. Borghaei, J. R. Brahmer, K. J. O'Byrne, W. J. Geese, P. Bhagavatheeswaran, S. K. Rabindran, R. S. Kasinathan, F. E. Nathan, S. S. Ramalingam, Nivolumab plus Ipilimumab in advanced non-small-cell lung cancer. *N. Engl. J. Med.* **381**, 2020–2031 (2019).

65. A. V. Serritella, N. K. Shenoy, Nivolumab plus ipilimumab vs nivolumab alone in advanced cancers other than melanoma: A meta-analysis. *JAMA Oncol.* **9**, 1441–1446 (2023).

66. A. El-Khoueiry, A. Bullock, A. Tsimberidou, D. Mahadevan, B. Wilky, P. Twardowski, B. Bockorny, J. Moser, W. O. Feliu, J. Grossman, K. Rosenthal, S. O'Day, M. Gordon, 479 AGEN1181, an Fc-enhanced anti-CTLA-4 antibody, alone and in combination with balstilimab (anti-PD-1) in patients with advanced solid tumors: Initial phase I results. *J. Immunother. Cancer* **9**, A509–A509 (2021).

67. T. Zhang, J. Sun, J. Li, Y. Zhao, T. Zhang, R. Yang, X. Ma, Safety and efficacy profile of mogamulizumab (Poteligeo) in the treatment of cancers: An update evidence from 14 studies. *BMC Cancer* **21**, 618 (2021).

68. F. A. Vargas, A. J. S. Furness, I. Solomon, K. Joshi, L. Mekkaoui, M. H. Lesko, E. M. Rota, R. Dahan, A. Georgiou, A. Sledzinska, A. B. Aissa, D. Franz, M. W. Sunderland, Y. N. S. Wong, J. Y. Henry, T. O'Brien, D. Nicol, B. Challacombe, S. A. Beers, T. C. Melanoma, T. C. Renal, T. C. Lung, S. Turajlic, M. Gore, J. Larkin, C. Swanton, K. A. Chester, M. Pule, J. V. Ravetch, T. Marafioti, K. S. Peggs, S. A. Quezada, Fc-optimized anti-CD25 depletes tumor-infiltrating regulatory T cells and synergizes with PD-1 blockade to eradicate established tumors. *Immunity* **46**, 577–586 (2017).

69. K. M. van Pul, J. C. L. Notohardjo, M. F. Fransen, B. D. Koster, A. G. M. Stam, D. Chondronasiou, S. M. Lougheed, J. Bakker, V. Kandiah, M. P. van den Tol, K. Jooss, R. Vuylsteke, A. J. M. van den Eertwegh, T. D. de Gruijl, Local delivery of low-dose anti-CTLA-4 to the melanoma lymphatic basin leads to systemic T_{reg} reduction and effector T cell activation. *Sci. Immunol.* **7**, eabn8097 (2022).

70. L. J. P. Van der Maaten, G. E. Hinton, Visualizing high-dimensional data using t-SNE. *J. Mach. Learn. Res.* **9**, 2579–2605 (2008).

71. N. Eling, N. Damond, T. Hoch, B. Bodenmiller, cytomapper: An R/Bioconductor package for visualization of highly multiplexed imaging data. *Bioinformatics* **36**, 5706–5708 (2020).

72. M. Zaw Thin, C. Moore, T. Snoeks, T. Kalber, J. Downward, A. Behrens, Micro-CT acquisition and image processing to track and characterize pulmonary nodules in mice. *Nat. Protoc.* **18**, 990–1015 (2022); https://doi.org/10.1038/s41596-022-00769-5.

73. J. H. Levine, E. F. Simonds, S. C. Bendall, K. L. Davis, E.-A. D. Amir, M. D. Tadmor, O. Litvin, H. G. Fienberg, A. Jager, E. R. Zunder, R. Finck, A. L. Gedman, I. Radtke, J. R. Downing, D. Pe'er, G. P. Nolan, Data-driven phenotypic dissection of AML reveals progenitor-like cells that correlate with prognosis. *Cell* **162**, 184–197 (2015).
